# STAT5B^N642H^ is a driver mutation for T cell neoplasia

**DOI:** 10.1172/JCI94509

**Published:** 2017-12-04

**Authors:** Ha Thi Thanh Pham, Barbara Maurer, Michaela Prchal-Murphy, Reinhard Grausenburger, Eva Grundschober, Tahereh Javaheri, Harini Nivarthi, Auke Boersma, Thomas Kolbe, Mohamed Elabd, Florian Halbritter, Jan Pencik, Zahra Kazemi, Florian Grebien, Markus Hengstschläger, Lukas Kenner, Stefan Kubicek, Matthias Farlik, Christoph Bock, Peter Valent, Mathias Müller, Thomas Rülicke, Veronika Sexl, Richard Moriggl

**Affiliations:** 1 Ludwig Boltzmann Institute for Cancer Research, Vienna, Austria.; 2 Institute of Animal Breeding and Genetics, University of Veterinary Medicine Vienna, Vienna, Austria.; 3 Institute of Pharmacology and Toxicology, University of Veterinary Medicine Vienna, Vienna, Austria.; 4 CeMM Research Center for Molecular Medicine of the Austrian Academy of Sciences, Vienna, Austria.; 5 Institute of Laboratory Animal Science, and; 6 Biomodels Austria (Biat), University of Veterinary Medicine Vienna, Vienna, Austria.; 7 IFA-Tulln, University of Natural Resources and Life Sciences, Tulln, Austria.; 8 Medical University of Vienna, Vienna, Austria.; 9 Center of Physiology and Pharmacology, Vienna, Austria.; 10 Center of Pathobiochemistry and Genetics, Institute of Medical Genetics, Medical University of Vienna, Vienna, Austria.; 11 Clinical Institute of Pathology, Medical University of Vienna, Vienna, Austria.; 12 Unit of Pathology of Laboratory Animals, University of Veterinary Medicine Vienna, Vienna, Austria.; 13 Max Planck Institute for Informatics, Saarbrücken, Germany.; 14 Department of Internal Medicine I, Division of Hematology and Hemostaseology, and; 15 Ludwig Boltzmann-Cluster Oncology, Medical University of Vienna, Vienna, Austria.

**Keywords:** Hematology, Oncology, Leukemias, Lymphomas, T cells

## Abstract

*STAT5B* is often mutated in hematopoietic malignancies. The most frequent *STAT5B* mutation, Asp642His (N642H), has been found in over 90 leukemia and lymphoma patients. Here, we used the *Vav1* promoter to generate transgenic mouse models that expressed either human STAT5B or STAT5B^N642H^ in the hematopoietic compartment. While STAT5B-expressing mice lacked a hematopoietic phenotype, the STAT5B^N642H^-expressing mice rapidly developed T cell neoplasms. Neoplasia manifested as transplantable CD8^+^ lymphoma or leukemia, indicating that the STAT5B^N642H^ mutation drives cancer development. Persistent and enhanced levels of STAT5B^N642H^ tyrosine phosphorylation in transformed CD8^+^ T cells led to profound changes in gene expression that were accompanied by alterations in DNA methylation at potential histone methyltransferase EZH2-binding sites. Aurora kinase genes were enriched in STAT5B^N642H^-expressing CD8^+^ T cells, which were exquisitely sensitive to JAK and Aurora kinase inhibitors. Together, our data suggest that JAK and Aurora kinase inhibitors should be further explored as potential therapeutics for lymphoma and leukemia patients with the STAT5B^N642H^ mutation who respond poorly to conventional chemotherapy.

## Introduction

The 2 signal transducer and activator of transcription 5 proteins, STAT5A and STAT5B, encoded by 2 different genes with 89% DNA sequence homology, are downstream targets of cytokines and growth factors (1, 2). STATs are highly expressed and/or hyperactivated by tyrosine and serine phosphorylation in numerous hematopoietic cancers (3–6). The 2 STAT5 proteins have been reported to have redundant functions largely due to overlapping genome binding sites (7–9). There are different phenotypes upon genetic loss or somatic point mutation resulting in hyperactivation of STAT5A or STAT5B. STAT5B has a dominant role in immune cells, as suggested by its higher expression levels compared with STAT5A in NK or T cell subsets (7–9). Interestingly, mutations in cancer patients have predominantly been found in the Src homology 2 (SH_2_) domain of human STAT5B (hSTAT5B). This suggests that differences in hematopoietic transformation are due to differences in the level of STAT5 proteins and possible distinct chromatin-remodeling capabilities as a result of interactions with other transcriptional regulatory proteins (10–12). Recently, the hSTAT5B^N642H^ mutation was described as a gain-of-function (GOF) mutation in leukemic patients that causes enhanced and prolonged tyrosine phosphorylation (13–27). This mutation is associated with a poor prognosis and an increased risk of relapse, despite the use of combination chemotherapy (13). The N642H mutation is located in the center of the STAT5B SH_2_ domain, the phosphotyrosine-binding domain that is essential for the formation of parallel STAT5 dimers and efficient nuclear translocation (1). STAT5B^N642H^ has been found in more than 90 patients with 7 types of aggressive leukemia or lymphoma including γδ T cell–derived lymphoma, hepatosplenic T cell lymphomas, large granular lymphocytic (LGL) leukemia, T cell acute lymphoblastic leukemia (T-ALL), T cell prolymphocytic leukemia, NK/T cell lymphoma, and enteropathy-associated T cell lymphoma (13–25).

Studies in mice have implicated STAT5 signaling in the expansion of CD8^+^ T cells as well as the development of lymphoblastic lymphoma (28). Nevertheless, there is no evidence of whether the hSTAT5B^N642H^ mutation is capable of driving the development and progression of leukemia. Drug-sensitivity tests on hSTAT5B^N642H^-expressing leukemic blasts from T-ALL patients indicated that the mutation provides a survival advantage in leukemic cells (17).

Epigenetic abnormalities are major drivers of hematopoietic malignancies. Mutations in Tet methylcytosine dioxygenase 2 (TET2) and DNA methyltransferase 3α (DNMT3A) affecting DNA methylation are frequently found in T cell lymphoma (29). TET1/2 was also shown to interact with STAT5 in Tregs that are strictly dependent on STAT5 because of direct regulation of the STAT5 target genes *FOXP3* and *CD25* (30). Interestingly, the *DNMT3A* gene was shown to be controlled by STAT5 in AML cells (31). Drugs interfering with epigenetic changes are powerful tools in cancer drug development and have found entry into therapeutic strategies (29). A key role of STAT5 is to support the process of histone acetylation and methylation in T cells, which was shown for the *TCR* locus (32, 33). Furthermore, the histone methyltransferase EZH2 and histone deacetylase 1 (HDAC1) were shown to be recruited via STAT5 binding (34, 35).

Here, we investigated the oncogenic potential of the hSTAT5B^N642H^ mutation compared with the nonmutated hSTAT5B using *Vav1*-driven transgenic mouse models. In contrast to WT hSTAT5B, moderate hSTAT5B^N642H^ expression levels triggered leukemia or lymphoma development, which manifested as a transplantable CD8^+^ T cell disease. Transcriptome and DNA methylome analyses illustrated profound changes in gene expression and reduced DNA methylation of potential EZH2-binding sites, with Aurora kinases being one of the most altered genes in hSTAT5B^N642H^-transgenic animals. In line with this, we found that Aurora kinase and JAK inhibitors were effective in blocking neoplastic T cell expansion and organ infiltration driven by hSTAT5B^N642H^. This suggested that inhibitors of Aurora kinases and JAK have potential as a treatment for patients suffering from hSTAT5^N642H^-driven lymphoma or leukemia.

## Results

### hSTAT5B^N642H^ is an activating mutation in hematopoietic cells.

**Figure 1:**
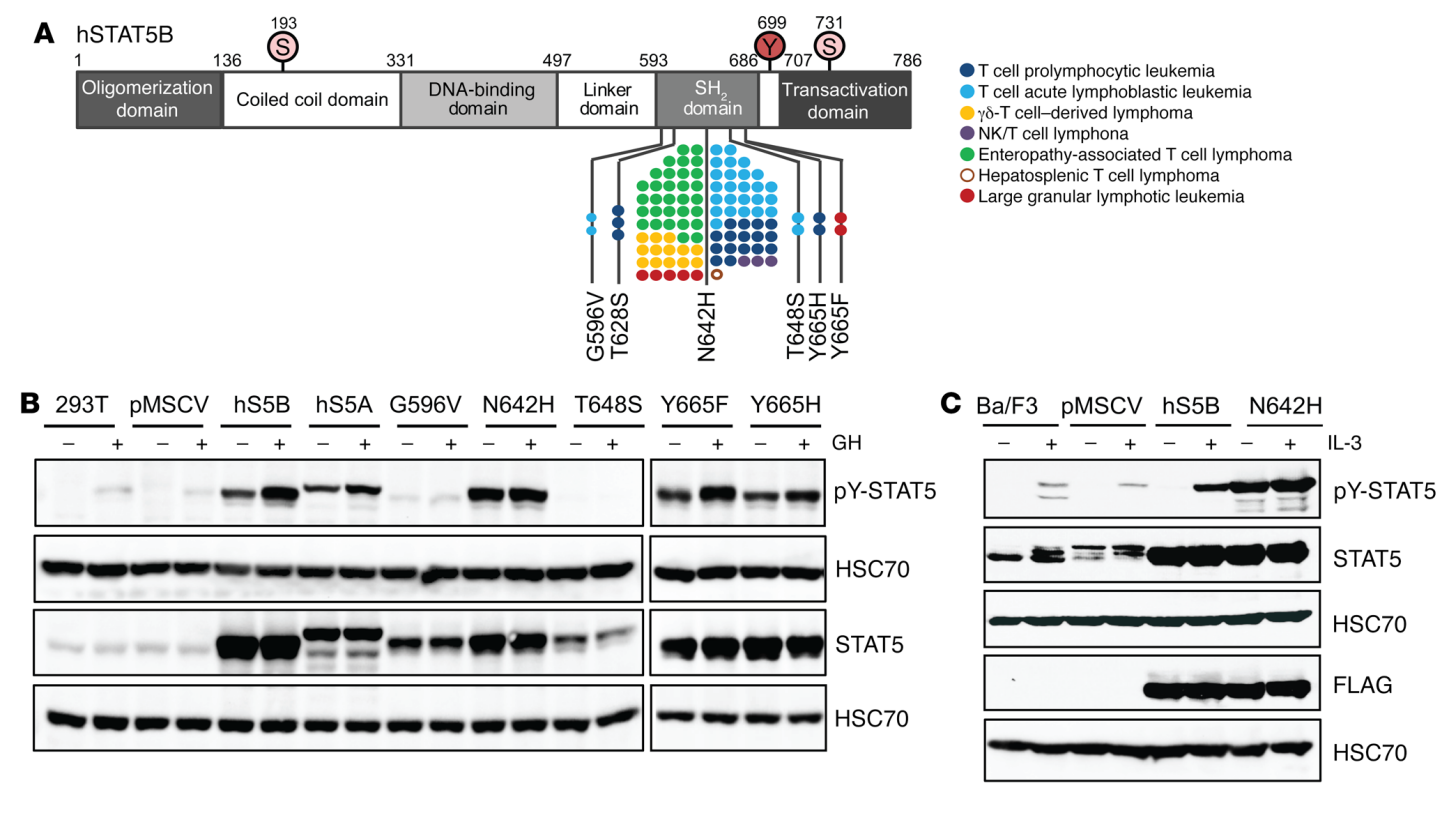
hSTAT5B^N642H^ is an activating mutation. (**A**) Schematic of STAT5B mutations identified in leukemia and lymphoma patients. Each dot represents 1 patient. (**B**) WB analysis of pY-STAT5, total STAT5 protein, and HSC70 in 293T cells that were transiently transfected with different hSTAT5B (hS5B) variants using a pMSCV-IRES-GFP vector, with or without growth hormone (GH) stimulation. (**C**) WB analysis of pY-STAT5, STAT5, FLAG, and HSC70 in hSTAT5B- or hSTAT5B^N642H^-expressing (N642H) Ba/F3 cells with or without IL-3 stimulation. (**B** and **C**) Nontransfected and pMSCV-transfected cells are shown as controls. Data presented in **B** and **C** are representative of 3 independent experiments. Samples were run on parallel gels for **B** and **C**, and a loading control is provided for each gel.

Somatic mutations of *STAT5B*, many of which are located in the SH_2_ domain, have been found in patients with lymphoid neoplasia (Figure 1A) (13–26, 36). To investigate the impact of these somatic mutations on hSTAT5B activity, we analyzed different potential GOF mutations in 293T cells using tyrosine phosphorylation of STAT5 (pY-STAT5) as a correlation for activity. We observed high pY-STAT5 levels under steady-state conditions in cells expressing the N642H mutation, the most frequent STAT5 mutation in patients with leukemia or lymphoma. The 2 SH_2_ domain variants Y665H and Y665F also showed enhanced activity in the absence of cytokine stimulation (Figure 1B). We observed a similar pattern of pY-STAT5B upon expression of the N642H mutant in the murine pro–B cell line Ba/F3 and the murine myeloid cell line 32D (Figure 1C). In contrast, expression of hSTAT5B at comparable levels failed to induce significant pY-STAT5 in the absence of IL-3 stimulation (Figure 1C and Supplemental Figure 1A; supplemental material available online with this article; https://doi.org/10.1172/JCI94509DS1). Importantly, hSTAT5B^N642H^ rendered Ba/F3 and 32D cells cytokine independent, supporting the finding of a proto-oncogenic potential of hSTAT5B^N642H^ (15) (Supplemental Figure 1B).

### Vav1-driven expression of hSTAT5B^N642H^ induces cancer.

**Figure 2:**
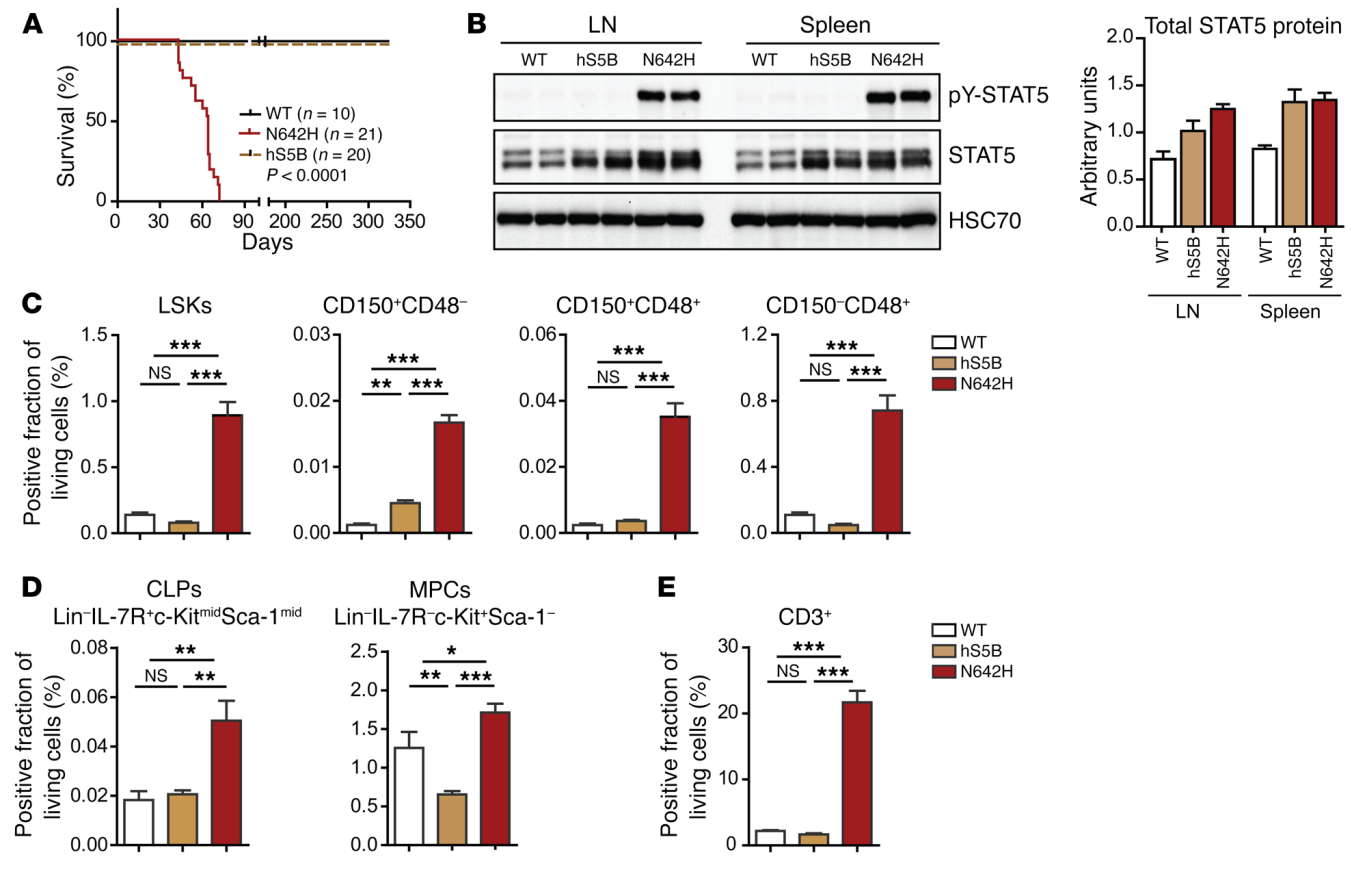
Moderate *Vav1*-driven expression of hSTAT5B^N642H^ in mice leads to HSC expansion. (**A**) Survival curve shows the percentages of disease-free survival of hSTAT5B^N642H^ (N642H) mice (*n* = 21) compared with that of hSTAT5B (hS5B) (*n* = 20) and WT (*n* = 10) mice. (**B**) WB analysis of pY-STAT5, total STAT5, and HSC70 in the LNs and spleens of WT mice and hSTAT5B^N642H^- and hSTAT5B-transgenic mice. Quantification of the WB was performed using ImageJ. Data are representative of 3 independent experiments. (**C**) Flow cytometric analysis of the percentage of LSKs, LT-HSCs (CD150^+^CD48^–^), ST-HSCs (CD150^+^CD48^+^), MPPs (CD150^–^CD48^+^), (**D** and **E**) common lymphoid progenitors (lineage^−^Sca1^+^IL-7R^+^AA4^+^), MPCs (lineage^−^Sca1^–^IL-7R^–^c-Kit^+^), and CD3^+^ cells in the BM of WT, hSTAT5B, and hSTAT5B^N642H^ mice. Analyses in **C**–**E** included 7-week-old WT (*n* = 7), hSTAT5B (*n* = 5), and hSTAT5B^N642H^ (*n* = 5) mice. Data represent the mean ± SD. *P < 0.05, **P < 0.01, and ***P < 0.001, by 1-way ANOVA with Bonferroni’s correction.

Given that hSTAT5B^N642H^ was found in different hematopoietic cancers, we expressed hSTAT5B or hSTAT5B^N642H^ in mice during early hematopoiesis using the *Vav1* oncogene promoter. This led to transgene expression primarily in cells of the hematopoietic system, including hematopoietic stem cells (HSCs) (37) (Supplemental Figure 2, A and B). Transgenic mice expressing hSTAT5B^N642H^ rapidly developed malignant disease leading to death between 40 and 100 days of age. hSTAT5B-transgenic mice showed no signs of disease when sacrificed at the age of 12 months or older (Figure 2A). Despite expressing comparable levels of total STAT5, only hSTAT5B^N642H^-transgenic mice showed elevated pY-STAT5 signals, indicating strong and persistent tyrosine phosphorylation (Figure 2B). In line with this observation, *Vav1*-driven expression of hSTAT5B^N642H^, but not hSTAT5B, led to increased numbers of progenitor cells throughout all early hematopoietic compartments, including lineage^–^Sca1^+^c-Kit^+^ cells (LSKs), long-term HSCs (LT-HSCs), short-term HSCs (ST-HSCs), and multipotent progenitors (MPPs) (CD150^+^CD48^–^, CD150^+^CD48^+^, CD150^–^CD48^+^ fractions) (Figure 2C). Likewise, the numbers of common lymphoid progenitors (CLPs) and myeloid progenitor cells (MPCs) were significantly elevated in mice expressing hSTAT5B^N642H^ (Figure 2D). hSTAT5B^N642H^ mice had 3 times more CLPs than did WT mice, which translated into expansion of CD3^+^ T cells, but not CD19^+^, B cells in their BM (Figure 2E and Supplemental Figure 2C). The elevated number of MPCs was also accompanied by a significant increase in the percentage of CD11b^+^Gr1^+^ cells in the BM of hSTAT5B^N642H^ mice (Supplemental Figure 2C).

**Figure 3:**
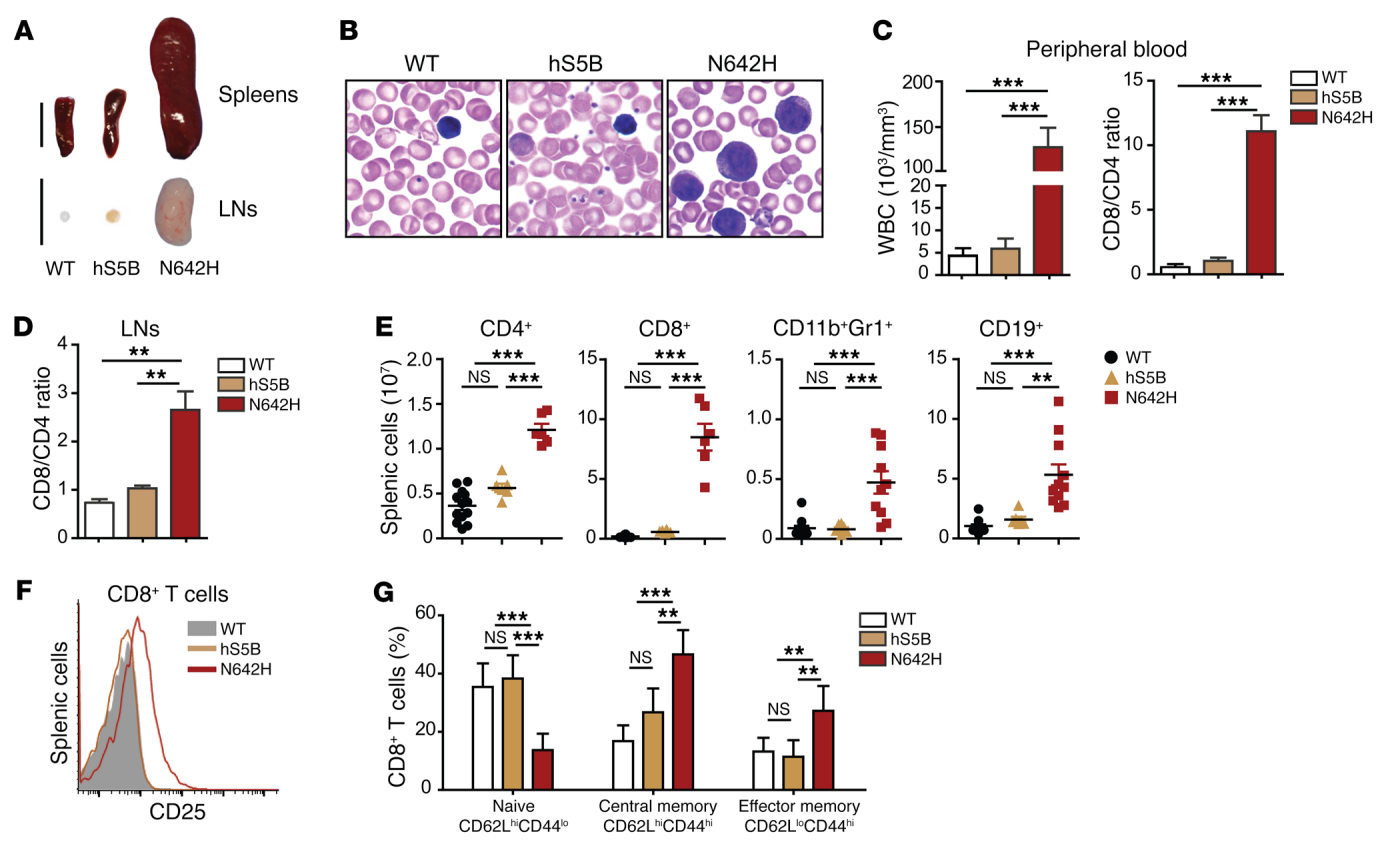
hSTAT5B^N642H^ mice suffer from an aggressive CD8^+^ T cell lymphoma. (**A**) Macroscopic comparison of hSTAT5B^N642H^ and hSTAT5B mouse spleens and LNs with those from WT mice. Scale bars: 1 cm. (**B**) Modified Wright staining of blood smears from hSTAT5B^N642H^ (N642H), hSTAT5B (hS5B), and WT mice (original magnification, ×100). (**C**) WBC count using an animal blood counter (scil Vet ABC). CD8/CD4 ratios in the peripheral blood were determined using flow cytometry. Analysis included 7- to 10-week-old WT (*n* = 20), hSTAT5B (*n* = 15), and hSTAT5B^N642H^ (*n* = 20) mice. (**D**) CD8/CD4 T cell ratios in LNs were determined using flow cytometry. Analyses included 7-week-old WT (*n* = 5), hSTAT5B (*n* = 5), and hSTAT5B^N642H^ (*n* = 5) mice. (**E**) Quantification of the absolute number of CD4^+^ and CD8^+^ T cells, myeloid cells (CD11b^+^Gr1^+^), and B cells (CD19^+^) in spleens from hSTAT5B^N642H^- and hSTAT5B-transgenic mice and WT mice. Analyses included 7-week-old WT (*n* = 13), hSTAT5B (*n* = 6), and hSTAT5B^N642H^ (*n* = 6 and 11) mice. (**F**) CD3^+^CD8^+^ splenic cells were analyzed by flow cytometry for their expression of CD25. Analyses included 8-week-old WT (*n* = 8), hSTAT5B (*n* = 9), and (*n* = 6) hSTAT5B^N642H^ mice. (**G**) CD3^+^CD8^+^ splenic cells were further analyzed for CD62L and CD44 expression. Analyses included WT (*n* = 8), hSTAT5B (*n* = 5), and hSTAT5B^N642H^ (*n* = 5) mice at 8 weeks of age. Data represent the mean ± SD. *n* ≥ 6. **P < 0.01 and ***P < 0.001, by 1-way ANOVA with Bonferroni’s correction.

Analysis of WBC counts in hSTAT5B^N642H^ mice revealed an increase of approximately 20-fold compared with that detected in hSTAT5B and WT mice (Figure 3C). The WBC count in hSTAT5B mice only increased slightly with age but remained within a physiological range (Supplemental Figure 3B). The drastic increase in the WBC count in STAT5B^N642H^ mice was correlated with an expansion of CD8^+^ T cells (Figure 3C). Similarly, CD8^+^ T cells increased by 3-fold in the lymph nodes (LNs) of hSTAT5B^N642H^ mice (Figure 3D), which was confirmed by immunohistochemical staining (Supplemental Figure 3C). The numbers of CD4^+^ T cells were also moderately increased, whereas the percentage, but not the total number, of CD19^+^ B cells was reduced in the LNs of hSTAT5B^N642H^ mice compared with controls (Figure 3E and Supplemental Figure 3D). Hematocrit levels were comparable in all mouse models (Supplemental Figure 3E). We also observed a mild expansion of other hematopoietic cell types such as CD19^+^ B cells, CD4^+^ T cells, and CD11b^+^Gr1^+^ myeloid cells in the spleen (Figure 3E and Supplemental Figure 3F).

**Figure 4:**
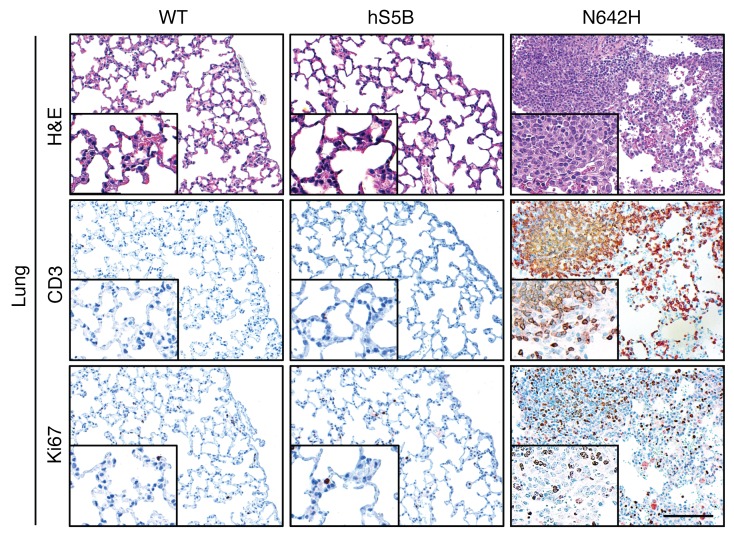
Highly proliferative T cells infiltrate into the peripheral organs of hSTAT5B^N642H^ mice. Histological analysis using H&E, CD3, and Ki67 staining of the lungs of 8- to 10-week-old hSTAT5B, hSTAT5B^N642H^, and WT mice. Data are a representative of 3 independent experiments. Scale bar: 100 μm. Original magnification, ×20 and ×40 (insets).

We used flow cytometry to analyze markers for T cell activation (CD25) and surface markers for T cell subpopulations, including naive CD8^+^ T cells (CD62L^hi^CD44^lo^), central memory CD8^+^ T cells (CD62L^hi^CD44^hi^), and effector memory CD8^+^ T cells (CD62L^lo^CD44^hi^) (38–40). This analysis showed that the leukemic cells expressed surface markers indicative of mature T cells with an activated phenotype and high expression of IL-2Rα (CD25), a direct target gene of STAT5 (41) (Figure 3F). Fifty percent of the diseased CD8^+^ T cells also expressed markers reminiscent of central memory T cells. Moreover, we found that the percentage of cells expressing markers for effector memory T cells was elevated in the diseased mice compared with that observed in WT controls (Figure 3G). High numbers of proliferating T cells were associated with splenomegaly and lymphoma formation, and proliferating T cells were found to heavily infiltrate peripheral organs, leading to fatal pulmonary obstruction (Figure 4 and Supplemental Figure 4).

**Figure 5:**
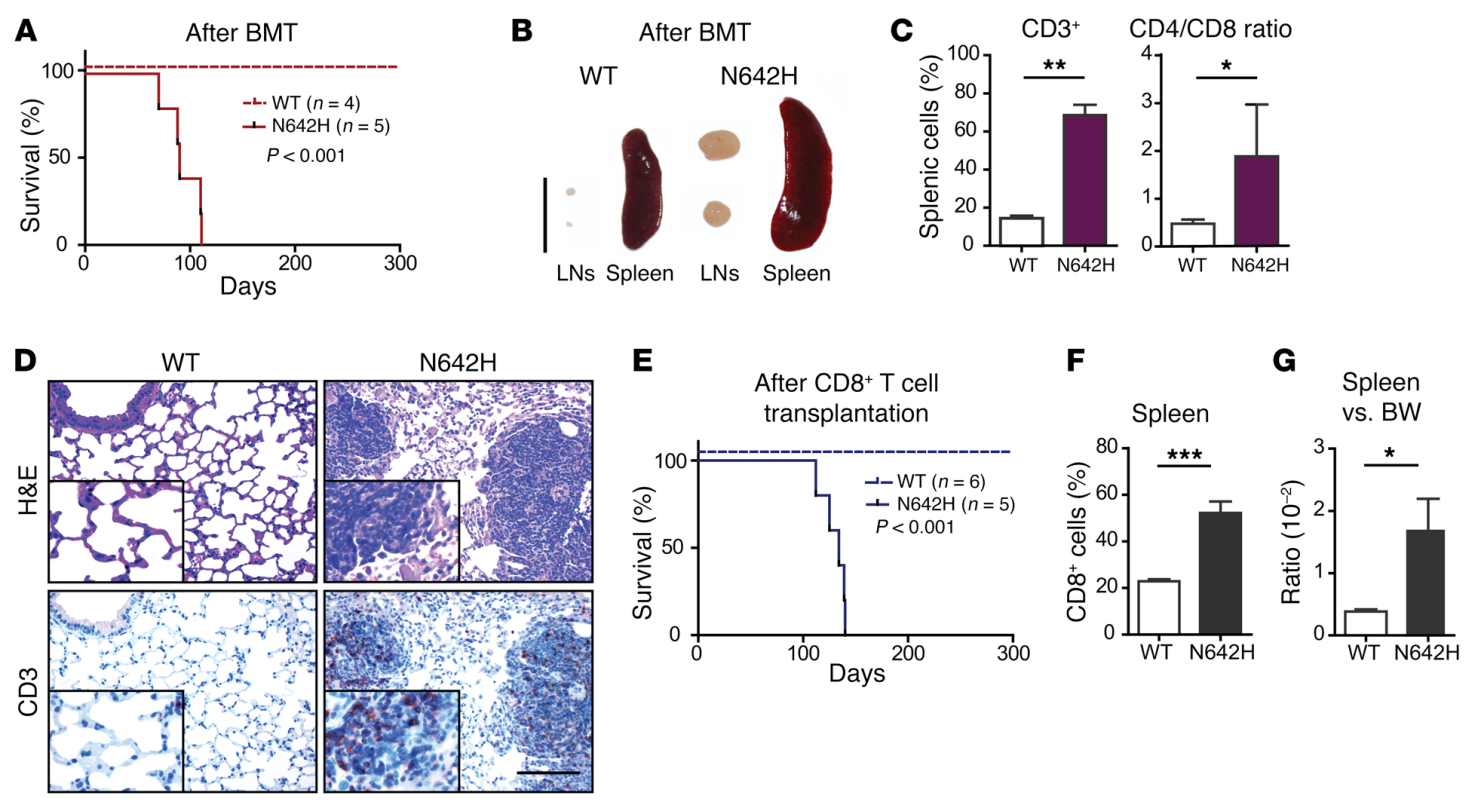
hSTAT5B^N642H^ CD8^+^ T cells are the cancer-initiating cells. (**A**) Percentage of disease-free survival following hSTAT5B^N642H^ whole BMT into 8-week-old NSG recipient mice compared with WT BMT. (**B**) Macroscopic view of LNs and spleen from a hSTAT5B^N642H^ BMT recipient mouse compared with those from a WT BMT recipient mouse. Scale bar: 1 cm. (**C**) Flow cytometric analysis shows the quantity of CD3^+^ cells and CD8/CD4 T cell ratio in the spleens of BMT recipient mice. (**D**) Histological analysis of CD3^+^ cells from the lungs of NSG recipient mice after hSTAT5B^N642H^ or WT BMT. Scale bar: 100 μm. Original magnification, ×20 and ×40 (insets). (**E**) Percentage of disease-free survival after hSTAT5B^N642H^ or WT CD8^+^ T cell transplantation into nonirradiated 8-week-old Ly5.1/CD45.1 recipient mice. (**F**) Flow cytometric analysis shows the quantity of splenic CD3^+^CD8^+^ cells in CD8^+^ T cell–transplanted mice. (**G**) Spleen versus BW ratios of WT and hSTAT5B^N642H^ CD8^+^ T cell–transplanted Ly5.1/CD45.1 mice. (**A**–**C**) *n* = 4 WT mice and *n* = 5 hSTAT5B^N642H^ mice; (**E**–**G**) *n* = 6 WT mice and *n* = 5 hSTAT5B^N642H^ mice. Data represent the mean ± SD. *P < 0.05, **P < 0.01, and ***P < 0.001, by unpaired, 2-tailed Student’s *t* test.

To test whether the T cell disease in hSTAT5B^N642H^-transgenic mice was transplantable, we transferred BM cells from mutant or WT control mice i.v. into nonirradiated, immunocompromised NSG recipient mice. The recipients of mutant cells became terminally sick approximately 3 months after injection (Figure 5A). Bone marrow transplantations (BMTs) induced disease, with a phenotype comparable to that of hSTAT5B^N642H^-transgenic mice. The disease was characterized by enlarged spleens and lymphoma formation, with T cell infiltration into peripheral organs (Figure 5B and Supplemental Figure 5, A and B) caused by excessive expansion and infiltration of CD8^+^ T cells (Figure 5, C and D). Of note, the i.v. injection of CD8^+^ T cells from diseased mice into nonirradiated Ly5.1^+^CD45.1^+^ recipient mice was sufficient to phenotypically recapitulate the primary disease, identifying the CD8^+^ T cells as the malignant cell pool (Figure 5, E–G, and Supplemental Figure 5, C and D).

### JAK inhibitors suppress disease progression in the hSTAT5B^N642H^-driven disease model.

**Figure 6:**
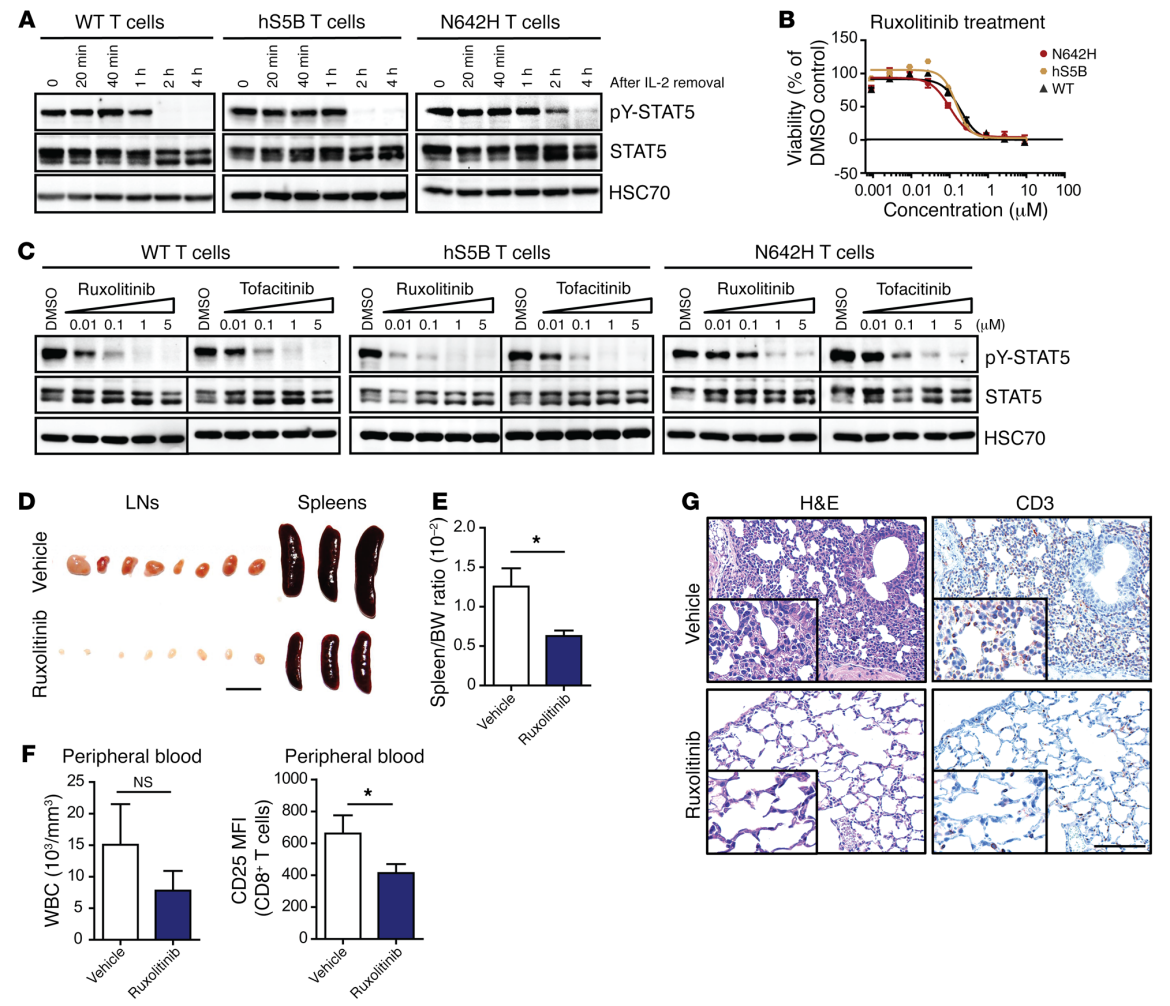
hSTAT5B^N642H^-driven diseased T cells can be treated with JAK inhibitors. (**A**) WB analysis of pY-STAT5 levels in isolated and cultivated LN T cells from hSTAT5B^N642H^, hSTAT5B, and WT mice after IL-2 removal. (**B**) Dose-response curve of WT, hSTAT5B^N642H^, and hSTAT5B T cells 72 hours after ruxolitinib treatment, analyzed using CellTiter-Glo (CTG) assay. IC_50_ values were determined using GraphPad Prism. Error bars indicate the mean ± SEM. DMSO (100% viability) and 10 μM bortezomib (0% viability) on each plate served as controls. (**C**) WB of hSTAT5B^N642H^, hSTAT5B, and WT T cell cultures after 5 hours of treatment with ruxolitinib or tofacitinib, analyzed for pY-STAT5. (**D**) Macroscopic view of LNs and spleens from CD8^+^ T cell–transplanted mice treated with ruxolitinib compared with vehicle controls. CD8^+^ T cell–recipient mice were treated with ruxolitinib at the dosage of 45 mg/kg twice a day for 30 days. (**E**) Quantification of spleen versus BW ratio of vehicle- and ruxolitinib-treated hSTAT5B^N642H^ CD8^+^ cell–transplanted mice. (**F**) WBC counts of vehicle- and ruxolitinib-treated hSTAT5B^N642H^ CD8^+^ cell–transplanted mice, measured using a scil Vet ABC animal blood counter. Flow cytometric analysis of CD25 expression in peripheral blood CD8^+^ T cells. MFI, mean fluorescence intensity. (**G**) Histological analysis of CD3^+^ cells in the lungs of recipient mice after treatment with ruxolitinib compared with the vehicle-treated group. Scale bar: 100 μm. Original magnification, ×20 and ×40 (insets). *n* = 5 vehicle-treated mice and *n* = 4 ruxolitinib-treated mice. Data represent the mean ± SD. *n* ≥ 6. *P < 0.05, by unpaired, 2-tailed Student’s *t* test. Data presented in **A**–**C** are representative of 3 independent experiments.

A number of treatment regimens have been suggested for leukemia and lymphoma patients carrying the hSTAT5B^N642H^ mutation. However, there is limited knowledge about the effectiveness of these treatments, partially because of the lack of a suitable preclinical model (17, 18). Typically, STAT5 is activated in response to cytokine signaling, and cells harboring the hSTAT5B^N642H^ mutant show prolonged pY-STAT5 levels upon stimulation rather than being constitutively active (18). When we analyzed the level of pY-STAT5 in primary T cells derived from the LNs of WT and hSTAT5B- and hSTAT5B^N642H^-transgenic mice, we detected drastically reduced levels of pY-STAT5 one hour after IL-2 deprivation in WT and hSTAT5B-expressing T cells. In contrast, low levels of pY-STAT5 remained detectable up to 4 hours after IL-2 removal in hSTAT5B^N642H^-expressing T cells (Figure 6A). The finding that cytokines efficiently activated hSTAT5B^N642H^ prompted us to test whether cells carrying the hSTAT5B^N642H^ mutation are sensitive to JAK inhibition. As expected, the FDA-approved JAK inhibitors ruxolitinib and tofacitinib reduced the activation of STAT5 and cell viability, with an IC_50_ of 0.11 μM (ruxolitinib) and 0.12 μM (tofacitinib) and comparable IC_50_ values for all genotypes (Figure 6, B and C, and Supplemental Figure 6A). Moreover, other FDA-approved drugs such as HDAC inhibitors for the treatment of T cell lymphoma were tested (42). Entinostat and several other drugs were also found to be effective in inducing apoptosis in T cells, with an IC_50_ in the nanomolar range, but did not exert differential effects between hSTAT5B- and hSTAT5^N642H^-expressing cells (Supplemental Figure 6A and Supplemental Table 1).

Following the in vitro data, we investigated the effect of ruxolitinib in vivo by treating hSTAT5B^N642H^ CD8^+^ T cell recipient Ly5.1^+^CD45.1 mice, 60 days after transplantation, with ruxolitinib (45 mg/kg) for a period of 30 days. The treatment significantly reduced the size of LNs and spleens (Figure 6, D and E). The WBC count as well as CD25 expression on donor hSTAT5B^N642H^ CD8^+^ T cells were also reduced upon ruxolitinib treatment (Figure 6F and Supplemental Figure 6B). Furthermore, ruxolitinib decreased the degree of T cell infiltration into the lungs, skin, BM, LNs, and spleens of treated mice, leading to a substantial reduction in disease burden (Figure 6G and Supplemental Figure 6C). The treatment did not significantly affect the myeloid cell population in the hematopoietic organs (Supplemental Figure 6D).

### hSTAT5B^N642H^ CD8^+^ T cells exhibit substantial changes in gene expression profile, accompanied by specific changes in DNA methylation.

**Figure 7:**
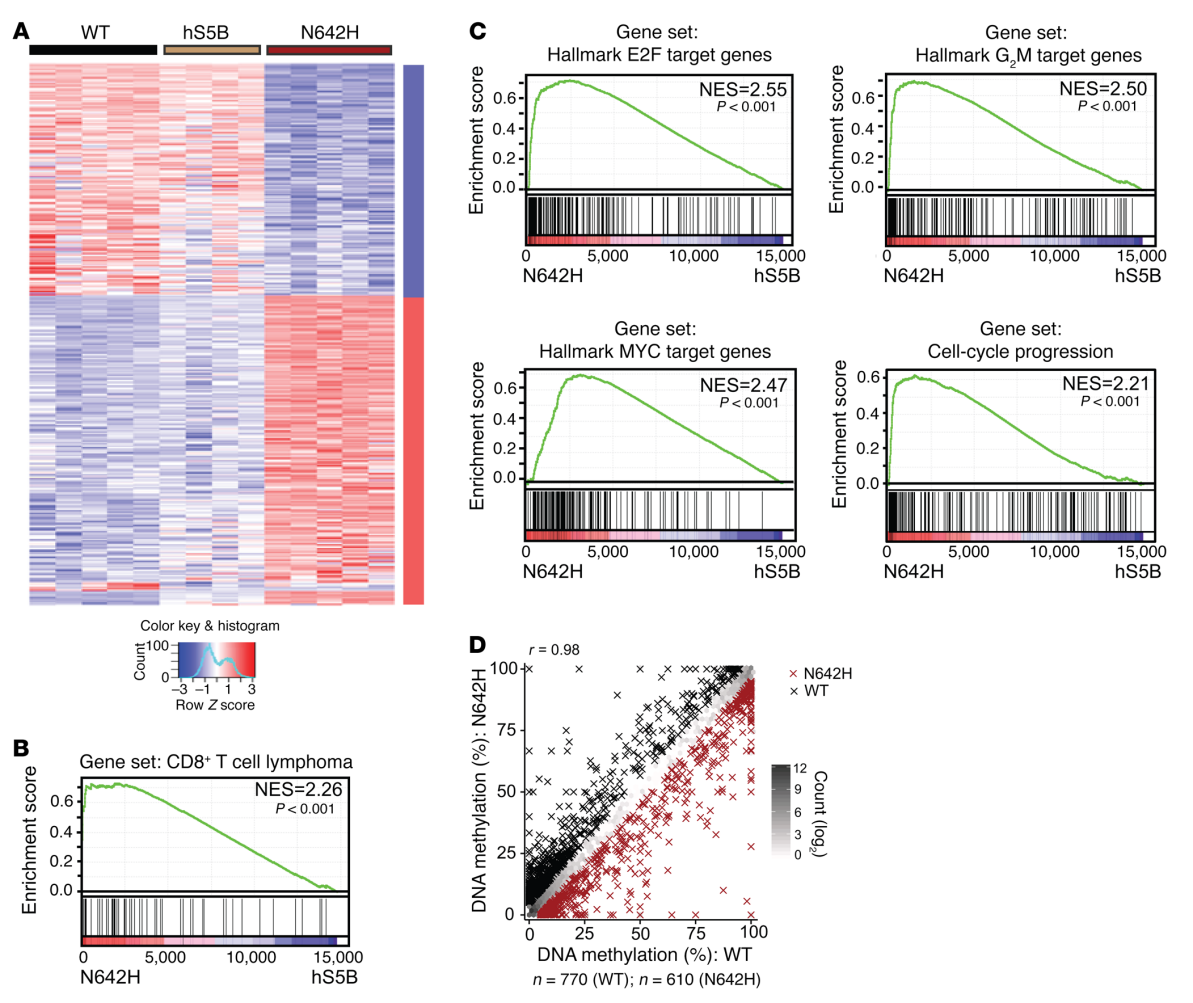
hSTAT5B^N642H^ provokes substantial changes in gene expression, accompanied by specific changes in DNA methylation of CD8^+^ T cells. (**A**) Heatmap showing *Z* scores of rlog-transformed and library size–normalized counts of genes upregulated (red) or downregulated (blue) in hSTAT5B or hSTAT5B^N642H^ and WT CD8^+^ T cells (FDR-adjusted *P* < 0.05). Analyses included 13-week-old WT (*n* = 5), hSTAT5B (*n* = 4), and hSTAT5B^N642H^ (*n* = 5) mice. Each column in the heatmap represents data from CD8^+^ T cells from 1 mouse of a given genotype, and each row represents data for a given gene. (**B**) Enrichment blot of the CD8^+^ T cell lymphoma expression signature. Barcode blot indicates the position of the gene in the gene set. Red and blue colors represent, respectively, positive and negative Pearson’s correlations with hSTAT5B^N642H^ CD8^+^ T cells. The gene set was obtained from published gene signature cytotoxic T cells (43, 44). (**C**) Top enriched gene sets are the results of GSEA including E2F target, G_2_M checkpoint, MYC target, and cell-cycle progression in hSTAT5B^N642H^ CD8^+^ T cells. *P* values in **B** and **C** were determined by Kolmogorov-Smirnov test. (**D**) Scatterplot contrasting the mean DNA methylation levels in WT and hSTAT5B^N642H^-mutant T cells in all CGIs covered in at least 1 sample per genotype (*n* = 15,209). The density of data points in each plot region is indicated by color intensity, and CGIs with lower DNA methylation in WT (*n* = 770) or hSTAT5B^N642H^ (*n* = 610) cells are indicated by black and red crosses, respectively (absolute difference ≥5 percentage points, *n* = 2 per genotype). Analyses included 13-week-old mice. NES, normalized enrichment score.

Given the leukemogenic effect of hSTAT5B^N642H^, which is not shared by WT hSTAT5B, we next investigated alterations in gene expression and epigenetic modifications in T cells derived from both mouse models. CD8^+^ T cells were isolated from the LNs of 13-week-old WT and hSTAT5B and hSTAT5B^N642H^ diseased mice, and mRNA sequencing analysis was performed. While the global expression patterns of WT and hSTAT5B CD8^+^ T cells were comparable, the gene expression signature of cells expressing hSTAT5B^N642H^ showed a distinct pattern (Figure 7A). We found a significant upregulation of 564 genes and a significant downregulation of 371 genes in T cells derived from hSTAT5B^N642H^ compared with that observed in WT T cells (FDR-adjusted *P* < 0.05) (Supplemental Figure 7A). As expected, known STAT5 targets such as *Ccl5*, *Ccr5*, *Pim1*, *Bcl2*, and *Il2r* were among the top upregulated genes, confirming *hSTAT5B^N642H^* transgene specificity (Supplemental Figure 7, B and C and Supplemental Tables 2 and 3) (7–9). Importantly, gene set enrichment analysis (GSEA) confirmed that genes upregulated in CD8^+^ T cell lymphoma patients were highly enriched, which emphasized the validity of our model (Figure 7B) (43, 44). Additional pathway analysis showed that E2F targets, the G_2_M checkpoint, and MYC targets were the most upregulated pathways, underlining the high proliferation rate of leukemic cells and indicating hSTAT5B^N642H^ as a driver for cell-cycle progression (Figure 7C and Supplemental Figure 7, D and E). In contrast, inflammatory gene pathways or developmental core cancer pathways were significantly downregulated (*P* < 0.05), as analyzed by pathway analysis (Supplemental Figure 7E).

Besides its function as a transcription factor, STAT5 can shape chromatin by interacting with other chromatin-remodeling enzymes such as EZH2 (35, 45). As changes in DNA methylation patterns have recently been associated with malignant disease and particularly with leukemia (46, 47), we questioned whether the dramatic changes observed in the expression profiles of hSTAT5B^N642H^ CD8^+^ T cells would also be reflected by alterations in the DNA methylome. Using reduced representation bisulfite sequencing (RRBS), we found that overall DNA methylation across CpG islands (CGIs) among hSTAT5B^N642H^ and WT T cells was highly consistent (Pearson’s *r* = 0.98), with only 1,380 CGIs being substantially different (absolute difference ≥5 percentage points) (Figure 7D) (48). When comparing WT and hSTAT5B CD8^+^ T cells, we found weaker differences and overlaps (Supplemental Figure 8A). Combining DNA methylation analysis with mRNA expression data, we identified a small set of genes with substantial and concordant changes in DNA methylation and expression of genes within the proximity of differentially methylated CGIs (Supplemental Figure 8, B and C). Interestingly, the genes with higher expression in hSTAT5B^N642H^ T cells and concordant loss of DNA methylation at nearby CGIs included the mitotic checkpoint protein KNTC1 (49) and the oncogene topoisomerase type IIα (*TOP2A*) (36, 50), which is known to regulate DNA topological structure and cell-cycle progression (Supplemental Figure 8B, right, sector II). None of these genes was substantially affected in hSTAT5B CD8^+^ T cells (Supplemental Figure 8B).

### Specific DNA methylation changes in hSTAT5B^N642H^ reveal targets for therapy.

**Figure 8:**
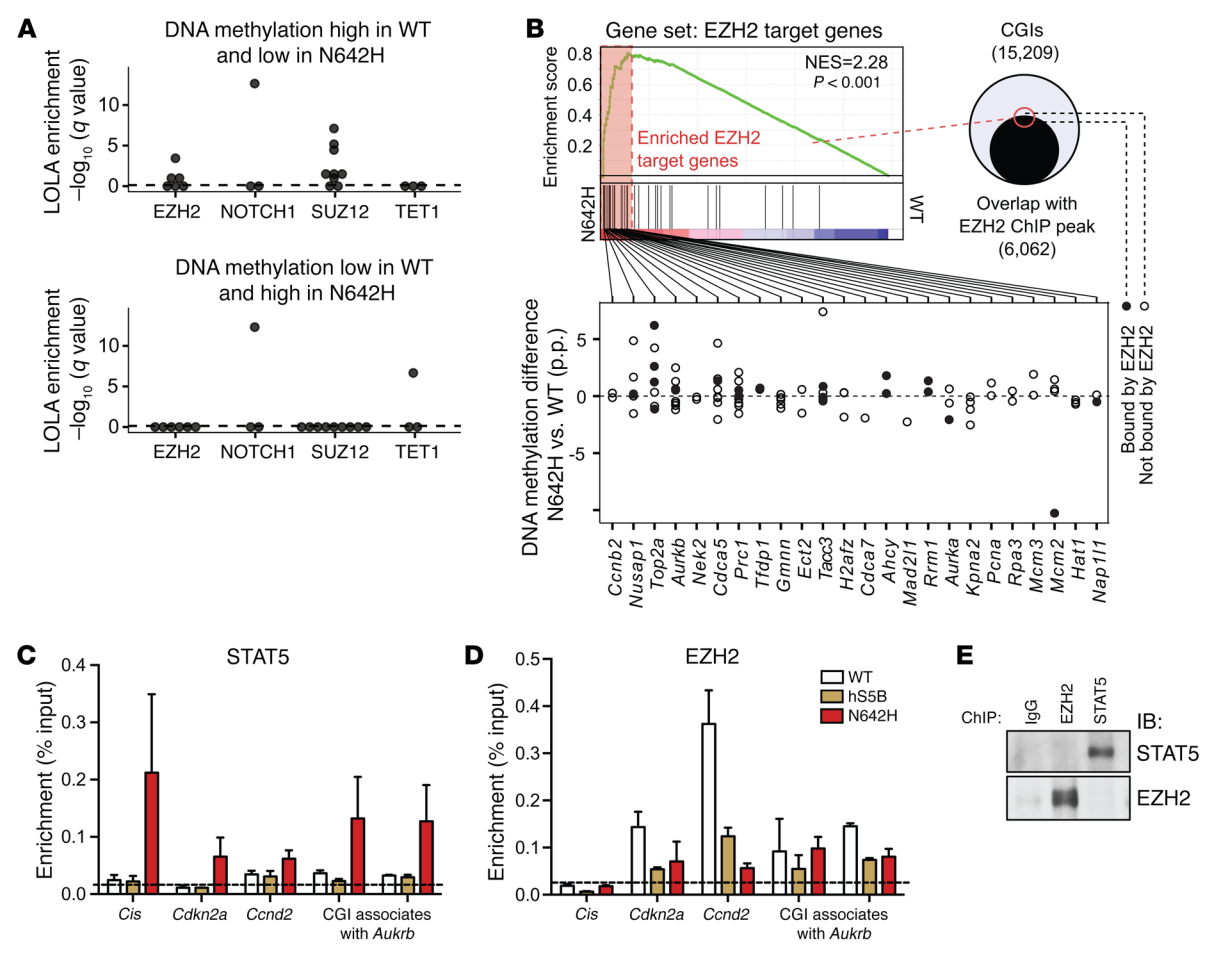
hSTAT5B^N642H^-driven DNA methylation changes accompanied by enhanced DNA-binding activity of STAT5 result in the induction of Aurora kinase B. (**A**) Region set enrichment analysis testing CGIs with lower DNA methylation in hSTAT5B^N642H^ cells than in WT cells (top) or lower DNA methylation in WT cells than in hSTAT5B^N642H^ cells (bottom). Enrichment was determined using LOLA (51). Each dot represents 1 ChIP-seq experiment for a given transcription factor from the CODEX database. The vertical dashed line represents the significance threshold (FDR-adjusted *P* ≤ 0.05). (**B**) Enrichment blot of EZH2 target genes in HSCs, together with their methylation states of EZH2-bound and EZH2-unbound CGIs 100 kb up- and downstream of the transcriptional start sites (TSSs). Barcode blot indicates the position of the gene in the gene set. Red and blue colors represent, respectively, positive and negative Pearson’s correlations with hSTAT5B^N642H^ CD8^+^ T cells. The gene set was obtained from the MSigDB (72). Black circles indicate CGIs overlapping with EZH2-binding sites. p.p., percentage points. *n* = 2 per genotype. ChIP with anti-STAT5 (**C**) or anti-EZH2 (**D**) in CD8^+^ T cells isolated from WT (*n* = 7), hSTAT5B (*n* = 7), or hSTAT5B^N642H^ (*n* = 4) mice. Binding of STAT5 to the *Cis* and *Ccnd2* promoters or binding of EZH2 to the promoter regions of *Cdkn2A* and *Ccnd2* served as positive controls. Horizontal dotted line indicates the threshold for nonspecific binding. (**E**) ChIP with anti-STAT5, anti-EZH2, or IgG in STAT5B^N642H^-expressing CD8^+^ T cells, followed by WB analysis. IB, immunoblot. Data presented in **C**–**E** are representative of 2 independent experiments. Error bars indicate the mean ± SD.

Location overlap analysis (LOLA) (51) of regions that lost methylation in T cells expressing hSTAT5B^N642H^ compared with WT cells revealed significant enrichment for sites known to bind EZH2 and/or SUZ12 proteins. These are components of polycomb repressor complex 2 (PCR2), which promotes methylation of histone 3 at lysine 27 (FDR-adjusted *P* ≤ 0.05, Fisher’s exact test) (Figure 8A, top, Supplemental Figure 9A, and Supplemental Table 4). STAT5 has recently been reported to oppose a network of transcription factors such as NF-κB and IKAROS in B cell acute lymphoblastic leukemia (52) and to interact with EZH2 (35). Furthermore, TOP2A expression has been previously linked to EZH2 expression in aggressive prostate cancer (53). Consistently, target genes of EZH2 and SUZ12 were found to be enriched in CD8^+^ T cells derived from hSTAT5B^N642H^ mice (Figure 8B, Supplemental Figure 9B, and Supplemental Table 5).

To investigate whether STAT5B^N642H^ has a role in the upregulation of these genes, we performed ChIP with isolated CD8^+^ T cells from WT, hSTAT5B, and hSTAT5B^N642H^ mice. Given its hyperactivation status, binding of hSTAT5B^N642H^ to DNA increased compared with that detected in WT murine STAT5B and hSTAT5B. The mutated STAT5 increased its binding to the *Cis* promoter and was also found at the promoter regions of the EZH2 known targets *Cdkn2a* and *Ccnd2*. In addition, it bound to the less methylated CGI in association with *Aurkb* (Figure 8C). Although EZH2 binding was found to be reduced in hSTAT5B^N642H^ CD8^+^ T cells, EZH2 retained its binding at the same CGI (Figure 8D and Supplemental Figure 9C). However, hSTAT5B^N642H^ was not shown to have direct interactions with EZH2 (Figure 8E).

### STAT5B^N642H^-expressing T cells are sensitive to AURKB inhibition.

**Figure 9:**
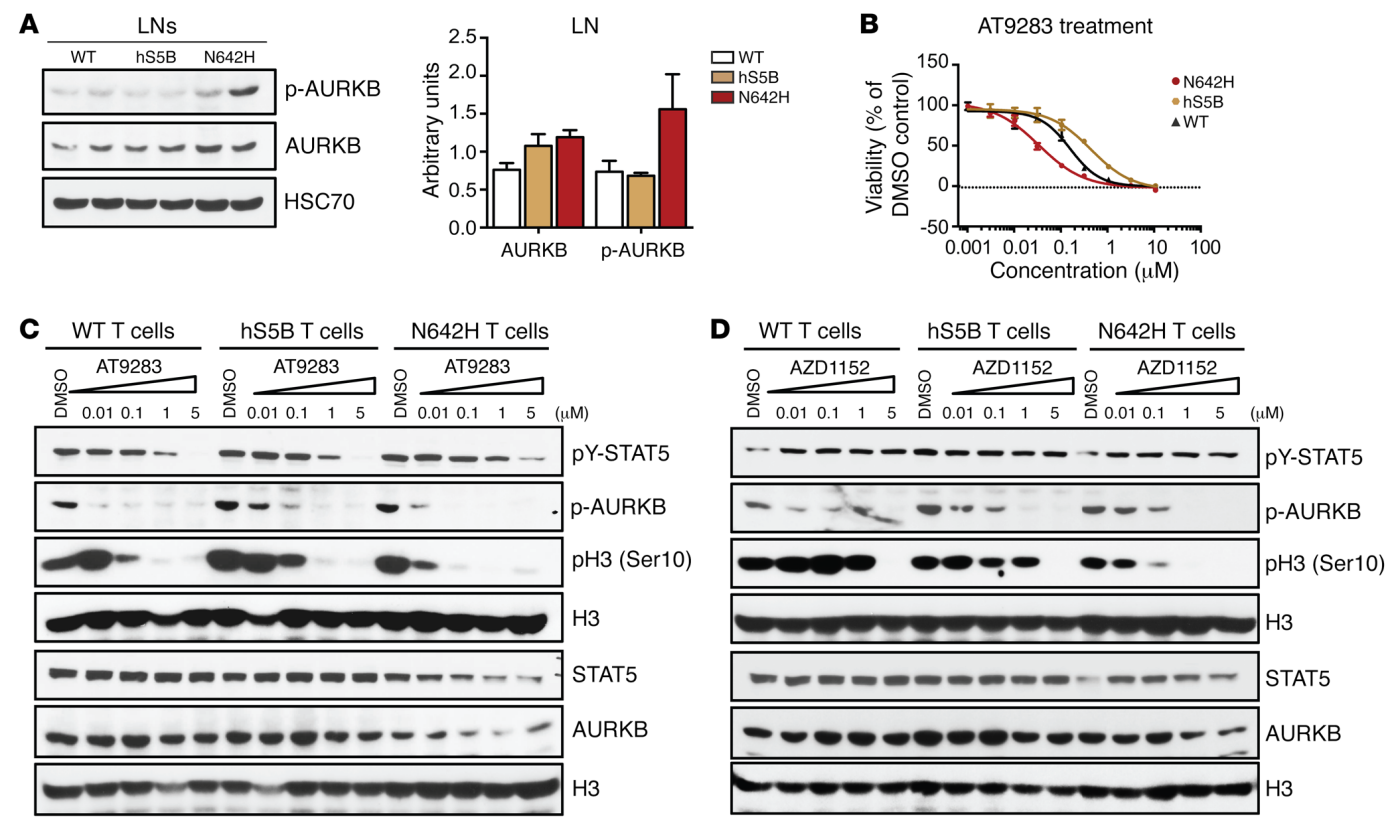
hSTAT5B^N642H^-driven diseased T cells are sensitive to Aurora kinase B inhibition. (**A**) WB analysis of p-AURKB, total AURKB, and HSC70 in LNs from WT and hSTAT5B^N642H^- and hSTAT5B-transgenic mice. WB quantification (bar graph) was performed using ImageJ. (**B**) Dose-response curves of WT, hSTAT5B^N642H^, or hSTAT5B T cells in response to AT9283 after 72 hours of treatment, analyzed using a CTG assay. IC_50_ values were determined using GraphPad Prism. Error bars indicate the mean ± SEM. DMSO (100% viability) and 10 μM bortezomib (0% viability) on each plate served as controls. *n* = 6 per genotype. (**C** and **D**) WB of hSTAT5B^N642H^, hSTAT5B, and WT T cell cultures after 5 hours of treatment with AT9283 or AZD1152, for determination of pY-STAT5, total STAT5, p-AURKB, total AURKB, p-H3 (Ser10), and total H3 levels. Data presented in **A**–**D** are representative of 3 independent experiments.

Among EZH2 target genes, the genes encoding Aurora kinase B (*Aurkb*) and DNA topoisomerase 2α (*Top2a*) were significantly upregulated, and AURKB targets were highly enriched in hSTAT5B^N642H^-expressing CD8^+^ T cells (Figure 8B and Supplemental Figure 10A). Western blot analysis showed that hSTAT5B^N642H^ mice had higher AURKB activity, and quantitative PCR (qPCR) analysis validated the hSTAT5B^N642H^-dependent upregulation of *Aurkb* levels in CD8^+^ T cells (Figure 9A and Supplemental Figure 10B). This led us to test the dual-specific JAK and Aurora kinase inhibitor AT9283 as a potential therapeutic in hSTAT5B^N642H^-expressing cells. We found that hSTAT5B^N642H^-expressing T cells were exquisitely more sensitive to AT9382, with a 10-fold lower IC_50_ compared with that of hSTAT5B-expressing T cells (Figure 9B), but not to etoposide, a TOP2A inhibitor (Supplemental Figure 10C). AT9283 was not effective in reducing STAT5 activation compared with ruxolitinib but efficiently reduced AURKB activity (Figure 9C). The high sensitivity of AT9283 could be an attribute of Aurora serine/threonine and JAK tyrosine/serine kinase combinatorial inhibition, as IC_50_ values of ruxolitinib and tofacitinib were similar in all genotypes (Figure 6 and Supplemental Figure 6). Combinatory treatment with ruxolitinib and AZD1152, an AURKB-specific inhibitor, showed an additive effect, which further supported our hypothesis (Supplemental Figure 10D). Although AZD1152 treatment did not affect STAT5 phosphorylation in all genotypes, it efficiently inhibited AURKB activity in hSTAT5B^N642H^-expressing T cells (Figure 9D).

## Discussion

Here, we provide evidence that the STAT5B^N642H^ mutation is a direct driver and not a bystander mutation for lymphoid malignancy. Expression of hSTAT5B^N642H^ triggers the development of leukemia or lymphoma characterized by highly proliferative and invasive CD8^+^ T cells. hSTAT5B^N642H^ activation remains largely cytokine dependent, which renders the diseased cells sensitive to JAK inhibition. When comparing T cells from transgenic hSTAT5B^N642H^ mice with those from their hSTAT5B counterparts, we found reduced DNA methylation of EZH2-binding sites. This correlated with an increase in the transcription of STAT5B and EZH2 target genes including the cell-cycle regulators *Top2A* and *Aurkb*, for which AURKB represents a potential therapeutic target.

T cells express considerably more STAT5B than do other cell types of the hematopoietic system (54–57), suggesting a privileged role for STAT5B in the T cell compartment (58). Moreover, STAT5B is the dominant STAT5 protein in effector and regulatory T cells, and the differences in STAT5A and STAT5B governing T cell function are largely associated with paralog expression differences (7, 59, 60). Transgenic mouse models expressing high levels of murine *Stat5a* or *Stat5b* developed lymphoblastic lymphoma at low penetrance (5%–25%) and with a late onset (up to 456 days) (28, 61).

We now show that moderate expression of hSTAT5B^N642H^, but not hSTAT5B, is sufficient to trigger an aggressive disease that causes rapid lethality at a young age, with full penetrance irrespective of gender, demonstrating the potent oncogenic role of the hSTAT5B^N642H^ mutation. Despite the *Vav1* promoter–dependent expression of hSTAT5B^N642H^ throughout the entire hematopoietic system, malignancy evolved in CD8^+^ T cells. This development could be a result of the CD8^+^ T cell sensitivity to the *Stat5a/b* gene dosage that was described previously in mice (62). Moreover, it has been reported that CD8^+^ T cells are more susceptible to oncogenic drivers, especially when these drivers are activated by cytokines or triggered via T cell receptors (62, 63). Similarly, hSTAT5B^N642H^ activation remains cytokine dependent, and the upregulation of IL-2Rα, a direct target of STAT5, resulted in CD8^+^ T cells becoming more sensitive to low doses of cytokine stimulation. So far, hSTAT5B^N642H^ mutations have primarily been found in patients with T cell or NK cell malignancies, pointing toward the sensitivity of these patients to aberrant STAT5 activation. When STAT5B^N642H^ was identified in CD8^+^ T cells in patients, such as those with T cell LGL (T-LGL) or epitheliotropic intestinal T cell lymphoma (13, 23, 64), it gave rise to more aggressive disease (26).

STAT5B^N642H^ has been shown previously to render Ba/F3 cells cytokine independent and to be constitutively active in HeLa cells (13, 15, 17). Ba/F3 cells have been used to determine the oncogenic potential of many leukemogenic drivers, however, the expression level of the oncogene is often very high, and the cells might have acquired additional mutations as a result of long-term cultivation. In cytokine-independent cell lines such as HeLa or HEK293T, STAT5 might be activated by other available growth stimuli. Cells expressing low levels of STAT5B^N642H^, however, remain dependent on cytokine stimulation, as shown in our diseased T cell model. This was also observed in NK cells by Küçük and colleagues (18).

The malignant transformation and expansion of CD8^+^ T cells in transgenic mice correlated with the upregulation of direct STAT5 target genes such as D-type cyclins, *Bcl2* family members, and *Pim* kinases, which promote cell-cycle progression and survival. Importantly, the most upregulated genes were E2F and MYC targets, which highlights the proliferative nature of the diseased T cells and explains the upregulation of numerous genes (65). STAT5B^N642H^ is hyperphosphorylated, and it would be interesting to study its potential different interactions with CD8^+^ T cell–specific activators or repressors compared with the less active WT STAT5B.

Recent work suggested that altered DNA methylation patterns in T cells are indicative or even causative for T cell transformation and that methylation of gene bodies was correlated with active transcription contributing to carcinogenesis (66, 67). Epigenetic regulators such as EZH2, TET1/2, and HDAC play important roles in leukemogenesis (68–71) and have been shown to interact with STAT5 (30, 31, 34, 35). EZH2 has been linked to the long-term repopulating capability, proliferation, and inhibition of apoptosis of HSCs (72, 73), all of which are important for transformed cells as well as for governing peripheral T cell fates (74). We demonstrate here that the expression of hSTAT5B^N642H^ not only led to transcriptional changes but also changed DNA methylation. Decreased methylation at EZH2- and SUZ12-binding sites in hSTAT5B^N642H^ T cells resulted in the upregulation of EZH2 target genes. There are conflicting reports regarding the interaction between EZH2 and STAT5. In 2011, Mandal and colleagues reported that STAT5 plays an essential part in the recruitment of EZH2 to repress Ig κ-chain (*Igk*) transcription in progenitor B cells (35). Others suggested that STAT5 and EZH2 compete for binding to regulatory sites, as shown in B cells and mammary epithelial cells (52, 75). We observed that, as a consequence of STAT5B hyperactivation, STAT5B^N642H^ bound more to DNA and subsequently upregulated many cell-cycle–regulating genes including *Top2a* and *Aurkb*. The fact that the cells were particularly sensitive to Aurora kinase inhibition underlines this observation.

Work by many groups identified STAT5 as an important target for therapy, since it is essential for JAK2^V617F^-, Flt3-ITD-, and BCR/ABL-driven diseases (76–78). Currently, intensive efforts are being made to inhibit STAT5 by blocking its SH_2_ domain (79). However, effective targeting of STAT5 remains challenging. Several different strategies have been suggested for the treatment of hSTAT5B^N642H^-expressing cells including the use of BCL2, MEK1/2, and JAK inhibitors (17, 18). Although some patients respond to JAK inhibitors, the lack of sensitivity in other patients requires broader therapeutic targets (16, 17). We believe that the hSTAT5B^N642H^-transgenic mouse model will serve as a valuable preclinical model. Using this model, we showed that the combined use of Aurora kinase and JAK inhibitors is a potential therapeutic strategy to treat lymphoma and leukemia patients with the STAT5B^N642H^ mutation.

We show here that hSTAT5B^N642H^ acts as a driver mutation in the development of leukemia and lymphoma and propose that upstream inhibition of JAK activation or the chromatin-remodeling partners of STAT5 could be an alternative targeting strategy for enhanced STAT5 activation.

## Methods

### Plasmid construction/mutagenesis and transfection.

hSTAT5B variants were generated using site-directed mutagenesis (80). Mutagenic PCR was performed using KOD Polymerase (Novagen). PCR products were subsequently digested with *DpnI* enzyme (New England BioLabs) to remove the methylated template according to the manufacturer’s protocol. *E. coli* was transformed with the digested reaction, and positive clones were selected by Sanger sequencing (81). Plasmid transfection was performed using Lipofectamine 2000 Reagent (Invitrogen, Thermo Fisher Scientific).

The cases of patients harboring the STAT5B^N642H^ mutation were assembled from previously published work (13–26, 36).

### Animals and generation of transgenic mice.

Transgenic mice were generated and bred on a C57BL/6NCrl background and maintained in a specific pathogen–free environment in the experimental mouse facility at the University of Veterinary Medicine (Vienna, Austria). We used the *Vav1-*hematopoietic vector *Vav1-hCD4 (HS21/45)* (37) to generate several transgenic mouse lines expressing hSTAT5B and hSTAT5B^N642H^ in the hematopoietic system and selected the lines B6N-Tg(STAT5B)731Biat and B6N-Tg(STAT5BN/H)726Biat, respectively, for further experiments. The hSTAT5B^N642H^ construct was generated using overlapping PCR technology as previously described (80) (forward primer: GAAAGAATGTTTTGGCATCTGATGCCTTTTAC; reverse primer: GTAAAAGGCATCAGATGCCAAAACATTCTTTC). The construct was digested with the *HindIII* restriction enzyme and gel purified for pronuclear injection (82). The transgenic mice were identified by genotyping PCR (forward primer: ACGCAGGACACAGAGAATGAG; reverse primer: GTGATGGTGGCGTTGACCTC). WT (C57BL/6NCrl) mice and B6-Ly5.1/Cr (B6.SJL-*Ptprc^a^Pepc^b^*/BoyCrCrl) mice were purchased from Charles River Laboratories, and NSG (NOD.Cg-*Prkdc^scid^ Il2rg^tm1Wjl^*/SzJ) mice were purchased from The Jackson Laboratory. Given the rapid development and strong phenotype of the hSTAT5B^N642H^-transgenic mice, the colony was propagated via in vitro fertilization with archived sperm cells (83).

### Hematocytometry and flow cytometry.

Mouse blood was collected into EDTA tubes (Greiner Bio-One Mini-Collect K3EDTA Tubes; Thermo Fisher Scientific) from the facial vein or from euthanized mice via cardiac puncture, and blood smears were stained using modified Wright staining. WBC counts were measured using an animal blood counter (scil Vet ABC).

For flow cytometry, erythrocytes were lysed using Gay’s solution (10 mM KHCO_3_ and 75 mM NH_4_Cl, pH 7.4). Single-cell suspensions were prepared by mincing organs through a 70-μm cell strainer (BD Biosciences). HSC staining was performed as previously described (84). All antibodies used for flow cytometry were purchased from eBioscience and BD (see Supplemental Table 6 for the list of the antibodies). All analyses were performed on the BD FACSCanto II using FACSDiva software (BD). Further analysis was performed using FlowJo software.

### Cell culture.

293T cells were cultivated with complete DMEM medium (10% FCS, 2 mM L-glutamine, 10 U/ml penicillin-streptomycin). Ba/F3 and 32D cells were cultivated with complete RPMI 1640 medium (10% FCS, 2 mM L-glutamine, 10 U/ml penicillin-streptomycin) (all from Gibco, Thermo Fisher Scientific) supplemented with IL-3 (1 ng/ml; ImmunoTools). IL-3 stimulation was performed with 10 ng/ml IL-3 for 20 minutes.

The hSTAT5B^N642H^, hSTAT5B, and B6N WT T cells were isolated from LNs and spleens from 8- to 12-week-old mice. Following T cell activation by anti-CD3 (BD), T cells were grown in complete RPMI 1640 medium containing 10 mM HEPES, 1× MEM nonessential amino acids, 50 μM β-mercaptoethanol (all from Gibco, Thermo Fisher Scientific), 1 mM sodium pyruvate (MilliporeSigma), and 100 U/ml human IL-2 (ProleukinÒ; Novartis).

Cytokine stimulation of T cells was performed with human IL-2 (100 U/ml; ProleukinÒ; Novartis), murine IL-4 (100 ng/ml; R&D Systems), or murine IL-7 (10 ng/ml; R&D Systems).

The 293T and 32D cell lines were gifts of M. Hengstschläger (Center of Pathobiochemistry and Genetics, Institute of Medical Genetics, Medical University of Vienna, Vienna, Austria) and F. Grebien (Ludwig Boltzmann Institute for Cancer Research, Vienna, Austria), respectively. The Ba/F3 cell line was provided by A. D’Andrea (Dana-Farber Cancer Institute, Harvard Medical School, Boston, Massachusetts, USA).

### Transplantation experiments.

BM cells (1 × 10^6^) from hSTAT5B^N642H^ or WT mice were transplanted by lateral tail vein injection into nonirradiated NSG mice. Mice were monitored daily and evaluated at the first sign of disease onset. CD8^+^ T cells were isolated using a CD8^+^ MagniSort Mouse T Cell Enrichment Kit (eBioscience), and sorted cells were checked with flow cytometry for their purity. Cells (1 × 10^6^) were injected i.v. into nonirradiated Ly5.1/CD45.1 mice.

### IHC.

Mouse organs were incubated overnight in 4% phosphate-buffered formaldehyde solution (Roti-Histofix; Carl Roth), dehydrated, embedded, and cut (4-μm-thick sections). For immunohistochemical staining, heat-mediated antigen retrieval was performed in citrate buffer at pH 6.0 (Dako) and stained with antibodies against CD3 (Thermo Fisher Scientific; RM-9107-S0; dilution 1:300); Ki67 (Novocastra, Leica Biosystem; NCL-Ki67p; dilution 1:1,000); and STAT5B (Santa Cruz Biotechnology; sc-1656; dilution 1:200) using standard protocols. Images were taken using a Zeiss Imager Z.1 microscope.

### Western blot analysis.

Western blotting (WB) was performed using standard protocols. The antibodies used were: monoclonal rabbit anti-mouse phosphorylated STAT5 (p-STAT5) (Invitrogen, Thermo Fisher Scientific; 716900; dilution 1:1,000); purified mouse anti-STAT5 (BD; 610191; dilution 1:2,000); monoclonal mouse anti-mouse HSC70 (Santa Cruz Biotechnology; sc-7298; dilution 1:10,000); monoclonal mouse anti-Flag M2 ( MilliporeSigma; F3156; dilution 1:1,000); monoclonal rabbit anti–p–Aurora A (Thr288), p–Aurora B (Thr232), and p–Aurora C (Thr198) (Cell Signaling Technology; 2914; 1:1,000); monoclonal rabbit anti-Aurora B/AIM1 (Cell Signaling Technology; 3094; 1:1,000); monoclonal rabbit anti–histone H3 (anti-H3) (Cell Signaling Technology; 4499; 1:1,000); monoclonal rabbit anti–p-H3 (Ser10) (Cell Signaling Technology; 53348; 1:1,000); ECL anti-mouse IgG; HRP-linked whole antibody from sheep (GE Healthcare; NA931V; dilution 1:10,000); ECL anti-rabbit IgG; and HRP-linked whole antibody from sheep (GE Healthcare; NA934V; dilution 1:10,000). WB quantification was performed using ImageJ software (NIH). (See the complete unedited blots in the supplemental material.)

### RNA sequencing and analysis.

mRNA was isolated from CD8^+^ T cells harvested from LNs from mice of all 3 genotypes. CD8^+^ T cells were enriched using a CD8^+^ MagniSort Enrichment Kit, and mRNA was isolated using TRIzol (MilliporeSigma) in combination with an RNeasy Mini Kit (QIAGEN). mRNA library preparation (SENSE mRNA-Seq Library preparation) and RNA sequencing (RNA-seq) was performed with an Illumina HiSeq 2500 at the Vienna Biocenter Core Facility (VBCF) Next-Generation Sequencing (NGS) Unit (www.vbcf.ac.at). Adapter trimming and removal of low-quality bases were performed using cutadapt. After alignment of reads against contaminating sequences (mitochondrial and ribosomal DNA), the remaining reads were aligned against GRCm37 using transcriptome-guided alignment with TopHat, version 1.4.1 (http://ccb.jhu.edu/software/tophat/index.shtml). Next, the htseq-count (http://htseq.readthedocs.io/en/master/count.html) with mode union was used to obtain gene counts for union gene models. Then, differentially expressed genes (log_2_ fold change >2 and FDR-adjusted *q* < 0.1) were determined using DESeq2, version 1.12.4 (Bioconductor).

For heatmaps, centered and scaled rlog-transformed library size–normalized counts were visualized using the heatmap.2 function of R package gplots, version 3.0.1 (https://www.rdocumentation.org/packages/gplots/versions/3.0.1).

Gene lists from differential expression analyses were ranked for the log_2_ fold changes between hSTAT5B^N642H^ and WT or hSTAT5B^N642H^ and hSTAT5B CD8^+^ T cells. Ranking lists were subsequently used for GSEA via the Broad Institute’s GSEAPreranked tool at the standard setting. Gene sets were obtained from current publications or from the Broad Institute’s Molecular Signatures Database (MSigDB). RNA-seq data and a description of the experimental design are available in the NCBI’s Gene Expression Omnibus (GEO) database (GEO GSE104557).

### RRBS and analysis.

Genomic DNA from purified CD8^+^ T cells was isolated using an AllPrep DNA/RNA Mini Kit (QIAGEN) and subsequently subjected to RRBS and analysis. RRBS was carried out as described earlier (85). In brief, 100 ng genomic DNA was digested for 12 hours at 37°C with 20 units of *MspI* (New England BioLabs; R0106L) in 30 μl of 1× NEB Buffer 2. Fill-in and A-tailing were performed by the addition of Klenow Fragment 3′→ 5′ exo- (New England BioLabs; M0212L) and dNTP mix (10 mM dATP, 1 mM dCTP, 1 mM dGTP). After ligation to methylated Illumina TruSeq LT v2 adaptors using Quick Ligase (New England BioLabs; M2200L), the libraries were size selected by performing a 0.75× clean-up with AMPure XP beads (Beckman Coulter; A63881). Up to 12 libraries were pooled in equal amounts on the basis of qPCR data and bisulfite converted using the EZ DNA Methylation Direct Kit (Zymo Research; D5020) with the following changes to the manufacturer’s protocol: the conversion reagent was used at ×0.9 concentration; incubation was performed for 20 cycles of 1 minute each at 95°C, followed by 10 minutes at 60°C; and the desulfonation time was extended to 30 minutes. Bisulfite-converted libraries were enriched for up to 17 cycles using PfuTurbo Cx Hotstart DNA Polymerase (Agilent Technologies; 600412). After a 2× AMPure XP clean-up, quality control was performed using a Qubit dsDNA HS Assay Kit (Life Technologies, Thermo Fisher Scientific; Q32854) and an Experion DNA 1K Analysis Kit (Bio-Rad; 700-7107). Sequencing was performed on an Illumina HiSeq 3000/4000 System using the 5-bp single-end mode. Initial data processing was carried out at the Biomedical Sequencing Facility of the Medical University of Vienna (http://www.biomedical-sequencing.at) using an in-house pipeline based on Pypiper (http://databio.org/pypiper) and Looper (http://databio.org/looper). Sequences were trimmed for adapters using Trimmomatic (86) with the ILLUMINACLIP settings “:2:40:7 SLIDINGWINDOW:4:15 MAXINFO:20:0.50 MINLEN:18.” All reads were aligned to the GRCm38 (mm10) assembly of the mouse genome using BSMAP in its RRBS mapping mode (87, 88). DNA methylation levels for individual CpGs were calculated using custom Python scripts and loaded into RnBeads (89) for exploratory analysis and to aggregate DNA methylation estimates per CGI. The aggregated values were loaded into R for further analysis. Differentially methylated regions (absolute difference ≥5 percentage points) were compared with ChIP-seq peaks from the CODEX database (90) using LOLA (51) to find significant overlaps (FDR-adjusted *P* ≤ 0.05) with potential regulators and effectors of DNA methylation differences. To compare DNA methylation at CGIs with genes, each CGI was associated with all genes within a 10-kb window after conversion of the gene coordinates to the mm10 reference genome using the UCSC LiftOver tool (https://genome.ucsc.edu/cgi-bin/hgLiftOver). RRBS sequencing data were deposited in the NCBI’s GEO database (GEO GSE104557).

### Viability assay.

Murine T cells (5 × 10^4^) from hSTAT5B^N642H^ and WT mice were seeded in triplicate in 96-well plates. JQ1, 5-azacytidine, entinostat, etoposide, AT9283, tofacitinib, and ruxolitinib (all from Selleckchem) were added and incubated for 72 hours. All compounds were solubilized in DMSO (MilliporeSigma). DMSO and bortezomib (Selleckchem) were used as a negative and positive control, respectively. CellTiter-Glo reagent (Promega) was used to determine viability, measured on an EnSpire plate reader (PerkinElmer). IC_50_ values were determined by nonlinear regression using GraphPad Prism 6 (GraphPad Software).

### In vivo ruxolitinib treatment.

hSTAT5B^N642H^ CD8^+^ T cell transplant recipients were treated with ruxolitinib (Chemietek) twice a day by oral gavage at a dosage of 45 mg/kg. Ruxolitinib was dissolved in DMSO (MilliporeSigma) and subsequently diluted in 0.5% methylcellulose (w/v) (MilliporeSigma).

### ChIP.

CD8^+^ T cells (10^7^ cells) from WT, hSTAT5B, and hSTAT5B^N642H^ mice were isolated using a CD8^+^ MagniSort Enrichment Kit. Isolated cells were washed twice with ice-cold PBS supplemented with inhibitors (1 mM Na_3_VO_4_, 1 mM NaF, 1× cOmplete Protease Inhibitor Cocktail [PIC], Roche) and fixed with DSG (2 mM, 30 min; Thermo Fisher Scientific). Cells were washed twice with cold PBS supplemented with inhibitor and fixed with formaldehyde (1%, 10 min; MilliporeSigma). Fixation was quenched by incubation with glycine (125 mM, 5 min; MilliporeSigma). T cells were subsequently harvested by centrifugation (350 *g*, 5 min). Cell lysis was performed with 1% SDS lysis buffer (1% SDS, 10 mM EDTA, 50 mM Tris [pH 8.1], 1 mM Na_3_VO_4_, 1 mM phenylmethylsulphonyl) at 4°C for 30 minutes and sonicated using a Diagenode Bioruptor (20 cycles with 30 seconds on, 30 seconds off, high magnitude). Sonication was followed by chromatin dilution (1:10) in dilution buffer (167 mM NaCl, 16.7 mM Tris [pH 8.1], 1.2 mM EDTA, 1.1% Triton-X, 0.01% SDS). Clear chromatin was harvested by centrifugation (10,000 *g*, 10 min, 4°C). Cleared chromatin was incubated with rolling at 4°C with 5 μg STAT5 (C-17) (Santa Cruz Biotechnology; sc-835 X), EZH2 (Diagenode; pAb-039-050), or IgG (Santa Cruz Biotechnology; sc-2027 X) overnight at 4°C. Diluted chromatin (1%) was kept as the input. Blocked Dynal Magnetic Beads (65 μl; Life Technologies, Thermo Fisher Scientific) were added per IP the following day and incubated for an additional 4 hours at 4°C. IP samples were washed 5 times with lithium chloride wash buffer (0.5 M LiCl_2_, 50 mM HEPES, 1 mM EDTA, 0.7% sodium deoxycholate, 1% NP-40) and then once in Tris-EDTA (TE) buffer containing 50 mM NaCl. Chromatin was eluted in 2× 100 μl elution buffer (1% SDS, 50 mM Tris, 10 mM EDTA). Eluted chromatin (20 μl) was used for WB analysis. Samples and inputs were incubated with 8 μl of 5 M NaCl at 65°C overnight and subsequently incubated with 0.5 M EDTA, 1 M Tris (pH 6.5), and proteinase K (10 mg/ml) for 2 hours at 55°C. RNA was lysed for 1 hour at 37°C using 0.2 mg/ml RNase-A (MilliporeSigma). Chromatin clean-up was performed using a PCR purification kit (QIAGEN). DNA was subjected to qPCR using GoTaq Real-Time qPCR (Promega), and the amount of amplification was quantified using standard curves. Primers are listed in Supplemental Table 7.

### Statistics.

Flow cytometric data are reported as the mean ± SD and were analyzed using GraphPad Prism 6 (GraphPad Software). Differences were assessed for statistical significance by an unpaired, 2-tailed Student’s *t* test and 1-way ANOVA with Bonferroni’s correction. Kaplan-Meier plots were analyzed using a log-rank (Mantel-Cox) test. *P* values for GSEA were determined using the Kolmogorov-Smirnov test. A *P* value of less than 0.05 was accepted as statistically significant.

### Study approval.

All animal experiments were approved by the institutional ethics committee and the Austrian Ministry BMWFW authorities under the animal license protocols BMWFW-68.205/0166-WF/V/3b/2015), BMWFW-68.205/0117-WF/V/3b/2016, and BMWFW-68.205/0103-WF/V/3b/2015. All mice were bred and maintained under standardized conditions at the University of Veterinary Medicine Vienna.

Additional details can be found in the Supplemental Methods.

## Author contributions

RM designed and supervised the study. HTTP, BM, MPM, EG, TJ, HN, ZK, TK, AB, SK, MF, MM, TR, VS, and RM designed and/or performed experiments. HTTP, RG, FH, and MPM analyzed data. JP and FG contributed to the interpretation of the data. LK interpreted IHC results. ME designed and performed experiments. PV, MH, and CB revised the manuscript with regard to critical intellectual content. HTTP, BM, VS, and RM wrote the manuscript.

## Supplementary Material

Supplemental data

## Version 1 12/04/2017

Electronic publication

## Version 2 01/02/2018

Print issue publication
