# Supplementary material for: STAT5BN642H is a driver mutation for T cell neoplasia
Source: J Clin Invest. 2017 Dec 4;128(1):387–401. doi: 10.1172/JCI94509 (PMC5749501; doi:10.1172/JCI94509)
Supplement: Supplemental data [file jci-128-94509-s001.pdf]

Supplemental methods

Cytospin

Ba/F3 and 32D cells were spun onto glass slides for 5 min at 800 rpm using CytoSpin III Cytocentrifuge (Shadon). The cells were stained with the “Hemacolor® Rapid staining of blood smear” kit (Merck) according to manufacturer’s instructions.

qPCR

mRNA was isolated from the cells using Trizol reagent (LifeTechnology). cDNA synthesis and qPCR were performed using RevertAid H Minus First Strand cDNA Synthesis Kit and 2xSYBR green Master Mix (Thermo Scientific), respectively. Primers used for qPCR were listed in Supplemental Table 5.

Electroporation

$5x10^6$ Ba/F3 or 32D cells were electroporated in 800 µl plain RPMI containing 20 µg of DNA. 32D cells were electroporated at 250 V and 960 µF and Ba/F3 cells were electroporated at 300 V and 960 µF. Following electroporation, cells were incubated with 20% FCS and 2 ng/ml IL-3 for 2 days.

Supplemental figure

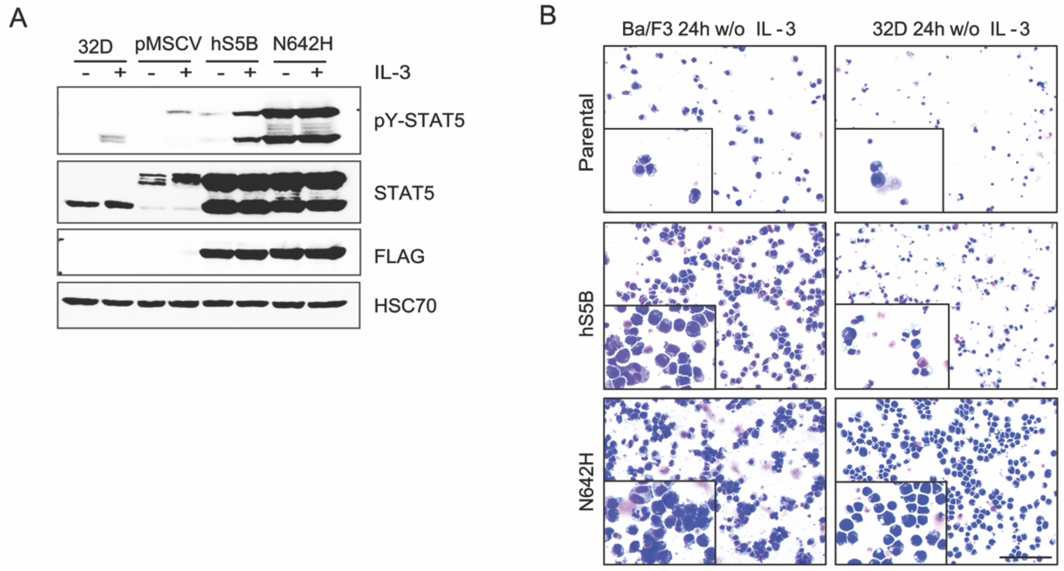

Supplement Figure 1

Supplemental Figure 1. hSTAT5B^{N642H} promotes cytokine-independent growth in lymphoid and myeloid cell lines. **(A)** 32D cells stably expressing hSTAT5B (hS5B) and hSTAT5B^{N642H} (N642H) were cultivated with and without IL-3. Western blot (WB) analysis of 32D cells showed hSTAT5B^{N642H} was tyrosine phosphorylated in the absence of IL-3. Parental and pMSCV-transfected 32D cells were used as controls. **(B)** Microscopic view of Hemacolor®-stained parental, hSTAT5B and hSTAT5B^{N642H} expressing Ba/F3 and 32D culture after 24h IL-3 starvation. Black bar denotes 100 µm. Data presented are representative of three independent experiments

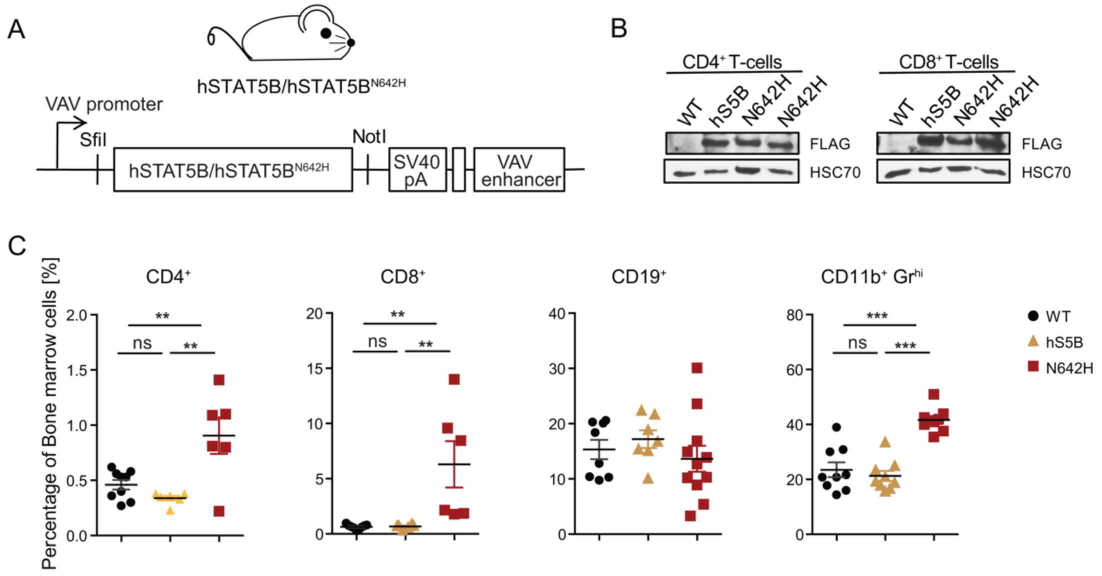

Supplemental Figure 2. hSTAT5B^{N642H} drives hematopoietic expansion *in vivo*. **(A)** Schematic of the *Vav1* vector used to generate two transgenic mouse models, hSTAT5B and hSTAT5B^{N642H}. **(B)** Western blot analysis of FLAG and HSC70 in CD4$^+$ T-cells and CD8$^+$ T-cells isolated from WT, hSTAT5B (hS5B) and hSTAT5B^{N642H} (N642H) mice**.** **(C)** The percentages CD3$^+$ CD4$^+$ T-cells, CD3$^+$ CD8$^+$ T-cells, myeloid cells (CD11b$^+$, Gr1$^+$) and B-cells (CD19$^+$) in the bone marrow (BM) of hSTAT5B^{N642H}, hSTAT5B transgenic mice and WT mice ($n \geq 5$, all 7 weeks-old) were quantified using flow cytometry. All data are presented as mean ± SD. **P* < 0.05; ***P* < 0.01; ****P* < 0.001 (1way ANOVA and Bonferroni corrected *P* value).

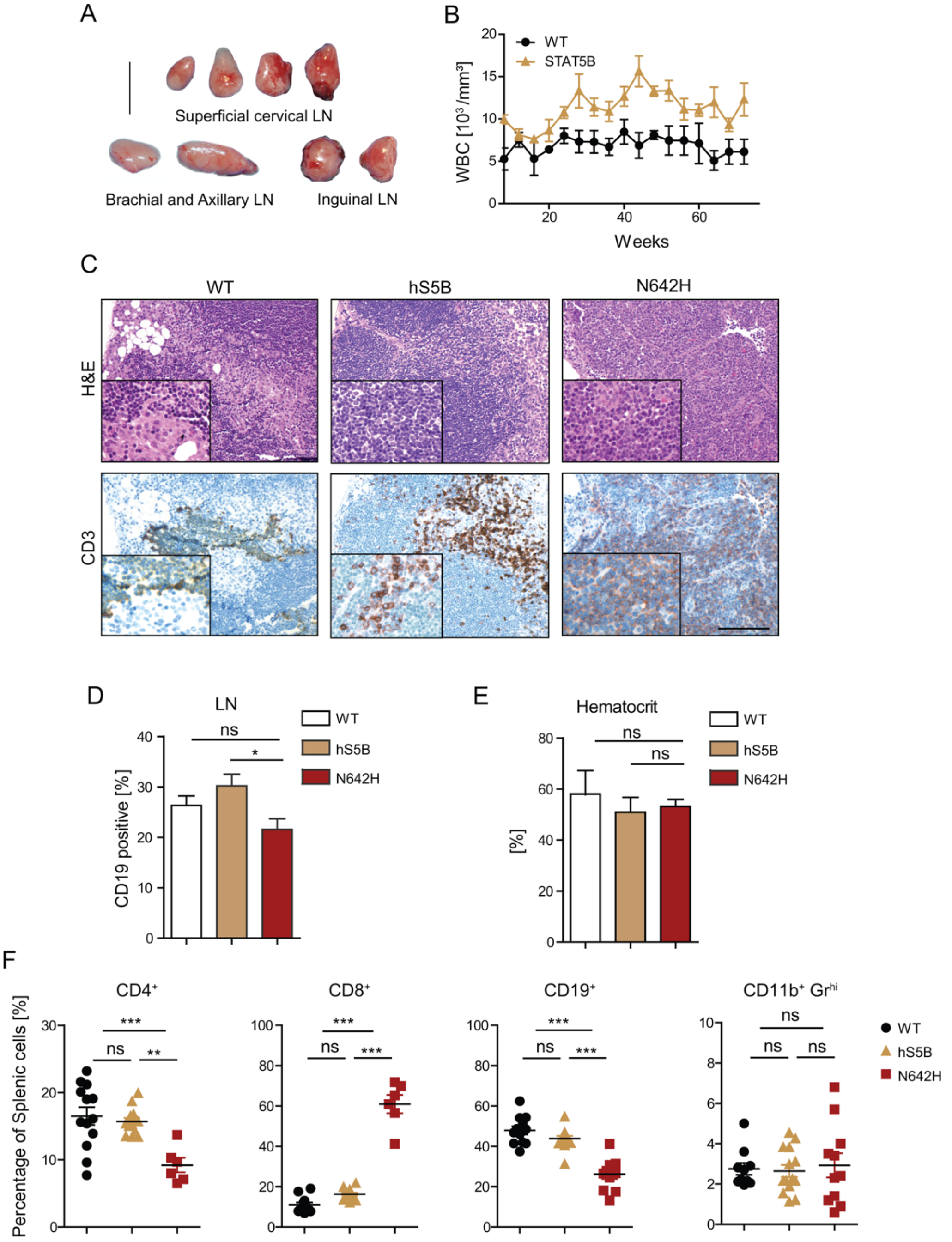

Supplemental Figure 3. hSTAT5B^{N642H} transgenic mice suffer from an aggressive form of leukemia/lymphoma. (A) Macroscopic view of superficial cervical, inguinal, brachial and axillary lymph nodes (LN) of 8 week-old hSTAT5B^{N642H} mice. Scale bar denotes 1 cm. **(B)** Quantification of white blood cell (WBC) count of WT (*n*=4) and hSTAT5B (hS5B) (*n*=8) mice over 80 weeks using

animal blood counter VetABC. **(C)** Histological analysis of $CD3^+$ cells in the LN of hSTAT5B, $hSTAT5B^{N642H}$ and WT mice showed severe T-cell infiltration in the LN of $hSTAT5B^{N642H}$ mice. Black bar denotes 100 µm. **(D)** The relative number of B-cells ($CD19^+$) in the LN of $hSTAT5B^{N642H}$ (N642H), hSTAT5B transgenic and WT mice were quantified using flow cytometry. **(E)** Quantification of percentage of hematocrit of WT and hSTAT5B 8 week-old mice using animal blood counter VetABC. n=8 WT and n=8 hSTAT5B, n=8 $hSTAT5B^{N642H}$. **(F)** The relative number of T-cell ($CD4^+$ or $CD8^+$), B-cells ($CD19^+$) and myeloid cells ($CD11b^+$, $Gr1^+$) in the spleen of $hSTAT5B^{N642H}$, hSTAT5B transgenic and WT mice were quantified using flow cytometry. n=13 WT and n=12 hSTAT5B, n>5 $hSTAT5B^{N642H}$. All data are presented as mean ± SD. *$P < 0.05$; **$P < 0.01$; ***$P < 0.001$ (1way ANOVA and Bonferroni corrected P value).

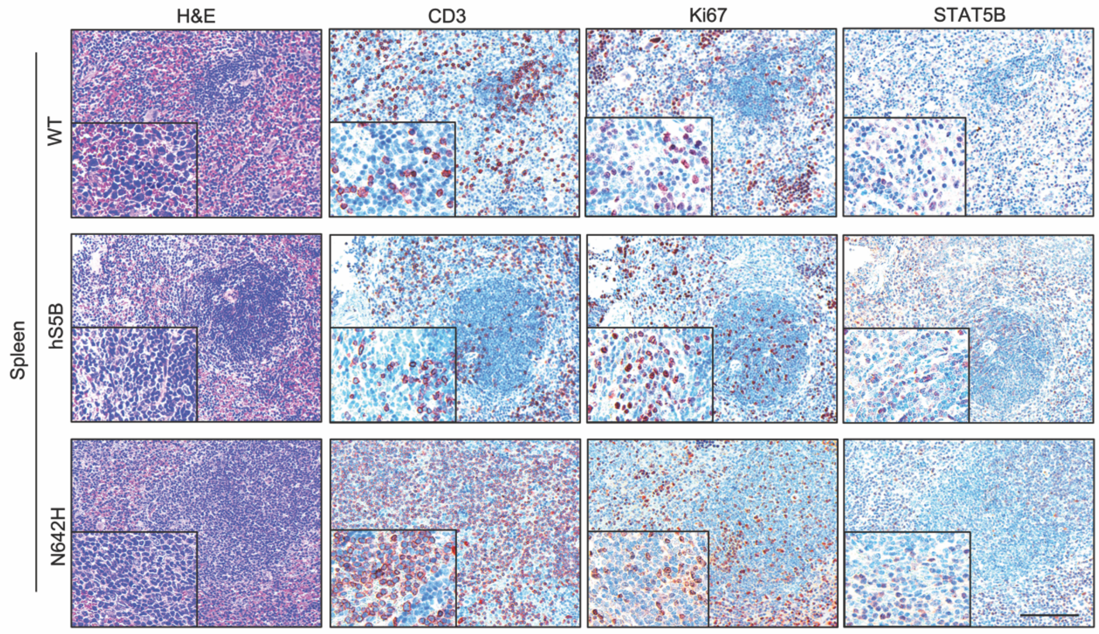

Supplemental Figure 4. Splenomegaly is caused by T-cell infiltration and proliferation in hSTAT5B^{N642H} transgenic mice. Histological analysis of the spleen from hSTAT5B (hS5B), hSTAT5B^{N642H} (N642H) and WT mice performing immunostaining with CD3, Ki76 and STAT5B specific antibodies. Black bar denotes 100 μm. All analyses were performed with 8 to 10-week-old mice. Data are representative of three independent experiments.

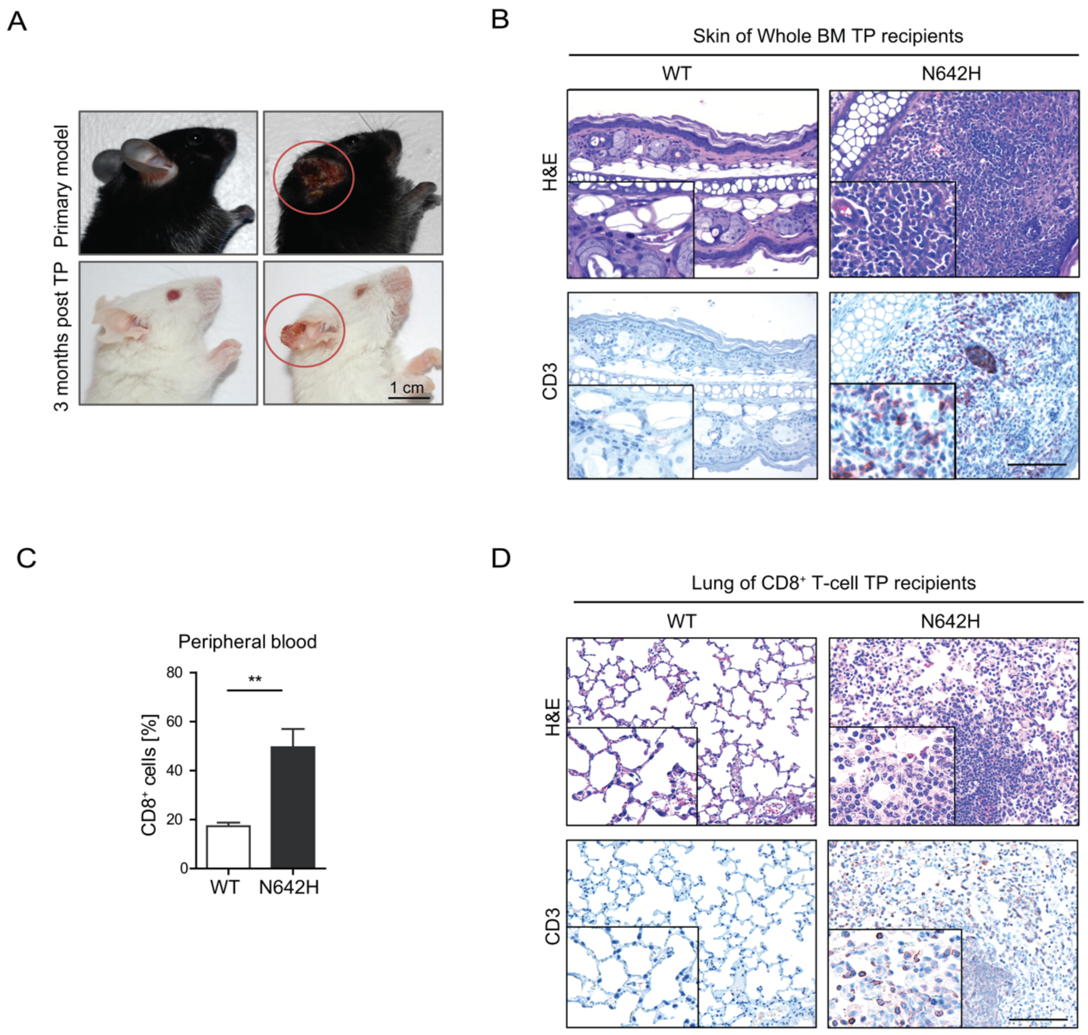

Supplemental Figure 5. hSTAT5B^{N642H} driven disease is transplantable. (A) Phenotypic representation of BM transplanted recipient in comparison to the primary disease model. **(B)** Histological analysis of the CD3^{+} cells in the skin of hSTAT5B^{N642H} whole BM transplanted NSG recipients. **(C)** Flow cytometry analysis showed the quantity of CD8^{+} T-cells in the peripheral blood of CD8^{+} T-cell transplanted Ly5.1/CD45.1 recipients. Black bar denotes 100 μm. In this analysis, n=6 WT and n=5 hSTAT5B^{N642H} recipients were used **(D)** Histological analysis of the CD3^{+} cells in the lungs of hSTAT5B^{N642H} (N642H) CD8^{+} T-cell transplant

recipients in comparison to the WT $CD8^+$ T-cell transplanted counter parts. Transplanted mice were analyzed at terminal state. Black bar denotes 100 μm. All data are presented as mean ± SD. *$P < 0.05$; **$P < 0.01$; ***$P < 0.001$ (unpaired two-tailed Student *t*-tests).

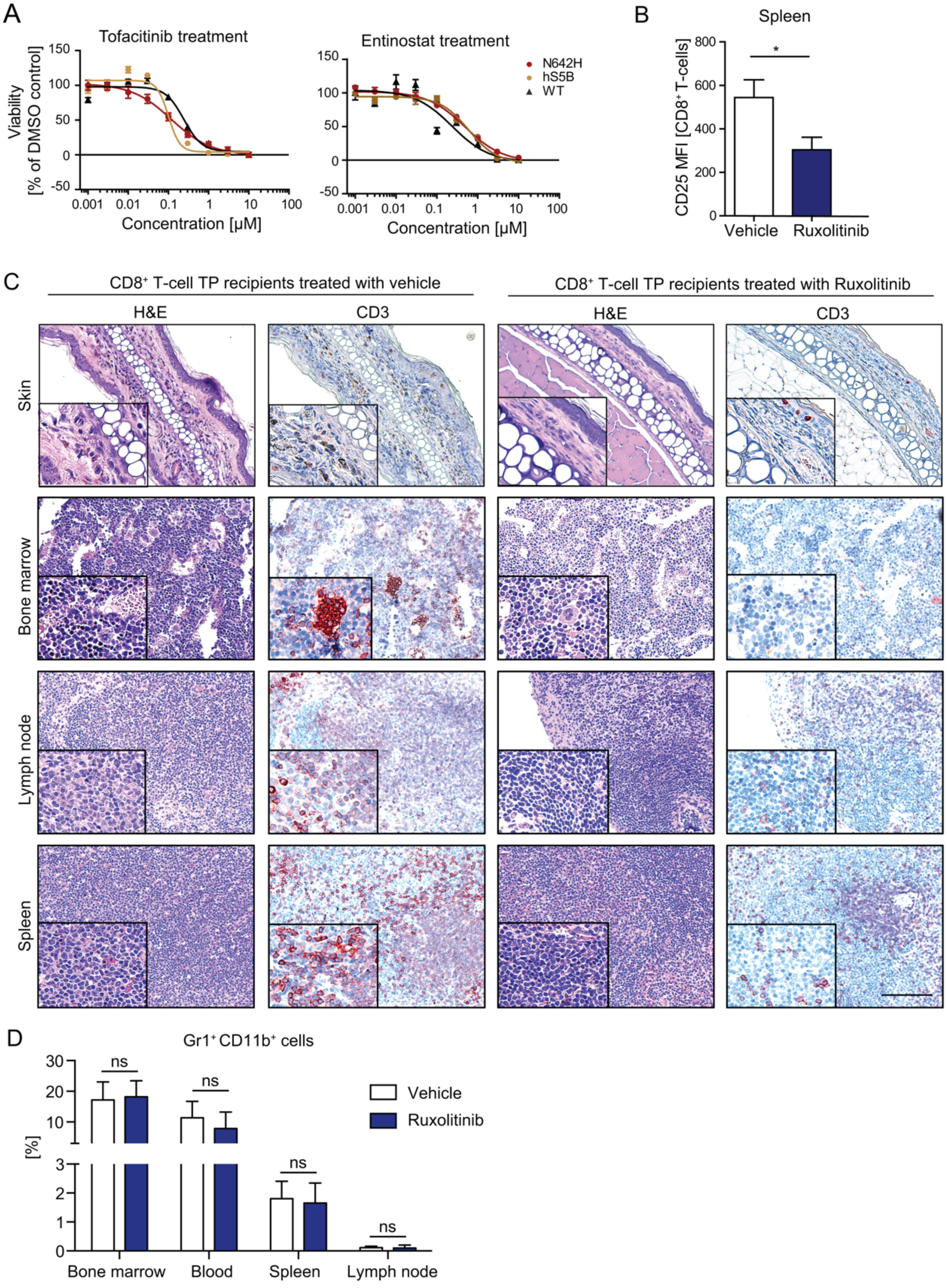

Supplemental Figure 6. Potential therapy for hSTAT5B^{N642H} driven T-cell disease. (A and B) Dose-response curves of WT, hSTAT5B (hS5B) and hSTAT5B^{N642H} (N642H) expressing T-cells in response to 72h treatment with JAK

inhibitors (left) and HDAC inhibitor entinostat (right) after 72h of treatment analyzed using CTG viability assay. IC_{50} values were determined using Graphpad Prism software. Error bars indicate ± SEM. **(B)** hSTAT5B^{N642H} CD8^{+} T-cells recipient mice were treated with ruxolitinib at the dosage of 45 mg/kg twice a day for 30 days. Flow cytometry analysis of CD25 expression on splenic CD8^{+} T-cells of hSTAT5B^{N642H} CD8^{+} T-cell transplanted recipient mice after ruxolitinib or vehicle treatment. **(C)** H&E and CD3 staining of the skin, bone marrow, lymph nodes and spleen of CD8^{+} T-cells transplanted mice after ruxolitinib treatment. **(D)** Flow cytometry analysis of Gr1^{+} CD11b^{+} cells in the blood, bone marrow, lymph nodes and spleen of CD8^{+} T-cells transplanted mice after ruxolitinib treatment. Black bar denotes 100 μm. $n \geq 5$. All data are presented as mean ± SD. *$P < 0.05$; **$P < 0.01$; ***$P < 0.001$ (unpaired two-tailed Student's *t* test).

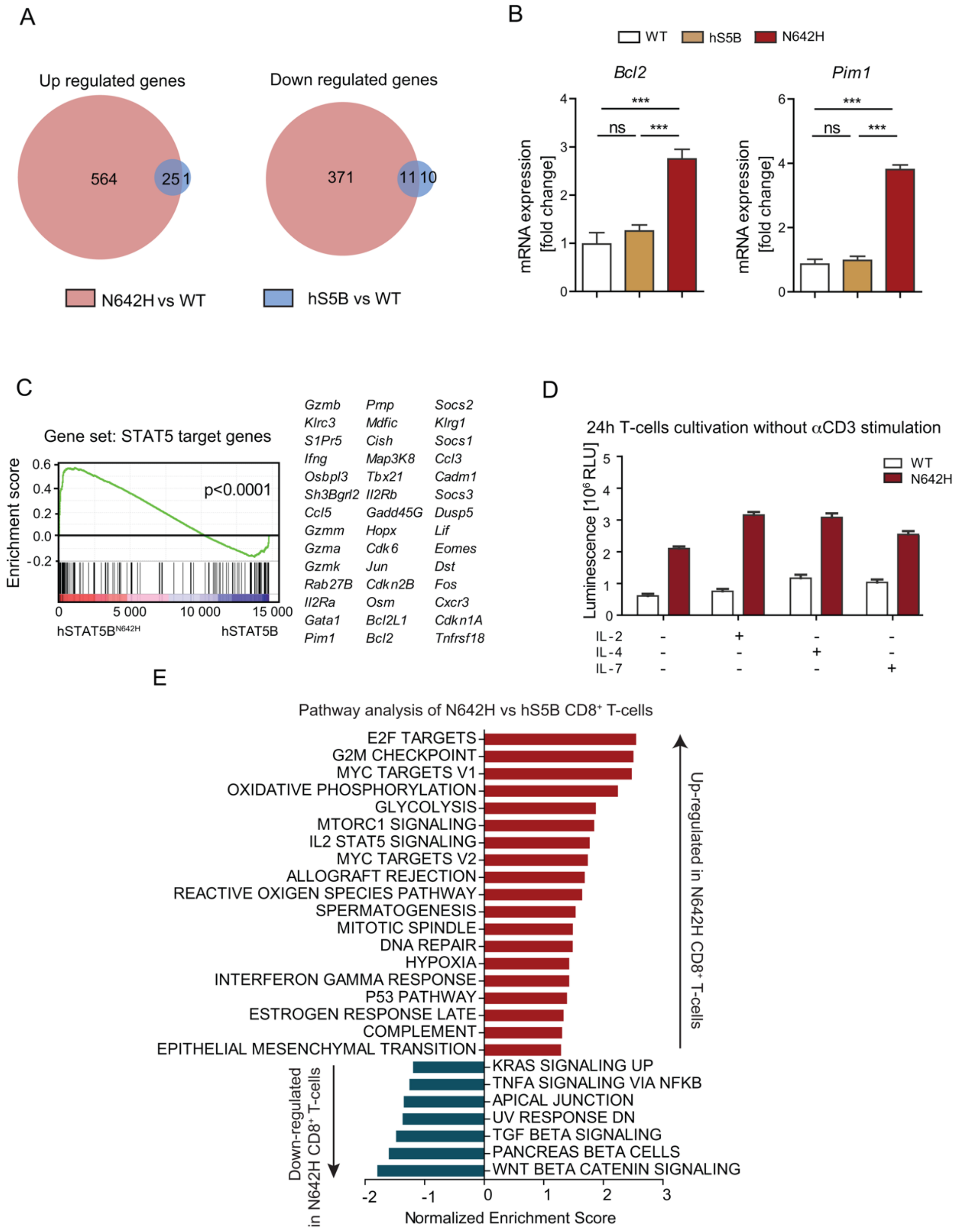

Supplemental Figure 7. The expression of hSTAT5B^{N642H} leads to the up-regulation of STAT5B target genes and cell cycle promoting genes. (A) Venn diagrams showing genes differentially regulated (more than 2-fold difference) in hSTAT5B (n=4) and hSTAT5B^{N642H} (n=5) expressing CD8$^+$ T-cells compared with

WT counterpart (n=5). **(B)** mRNA expression of *Bcl2* and *Pim1* in purified $CD8^+$ T-cells of 8 week-old WT, $hSTAT5B^{N642H}$ (N642H) and hSTAT5B (hS5B) transgenic mice were quantified using quantitative PCR. $n \geq 3$. **(C)** GSEA of genes that were ranked by signal/noise ratio according to their differential expression between hSTAT5B and $hSTAT5B^{N642H}$ $CD8^+$ T-cells. STAT5 target gene set are marked as vertical bars, and the enrichment score is shown in green. The list of significantly up-regulated STAT5 target genes was given using GSEA tool. **(D)** Comparison of T-cell viability between T-cells isolated form the LN of WT or $hSTAT5B^{N642H}$ mice under IL-2, IL-4 or IL-7 stimulation. Viability was measured with CTG viability assay. T-cells were isolated from 7 week-old mice and $n=3$ per genotype. **(E)** Top enriched gene sets associated with up or down-regulated genes in $hSTAT5B^{N642H}$ $CD8^+$ T-cells sorted by normalized enrichment scores obtained by GSEA tool using hallmark gene sets. All analyses were performed using 13 week-old mice. All data are presented as mean ± SD. $n \geq 6$. $*P < 0.05$; $**P < 0.01$; $***P < 0.001$ (unpaired two-tailed Student t-tests).

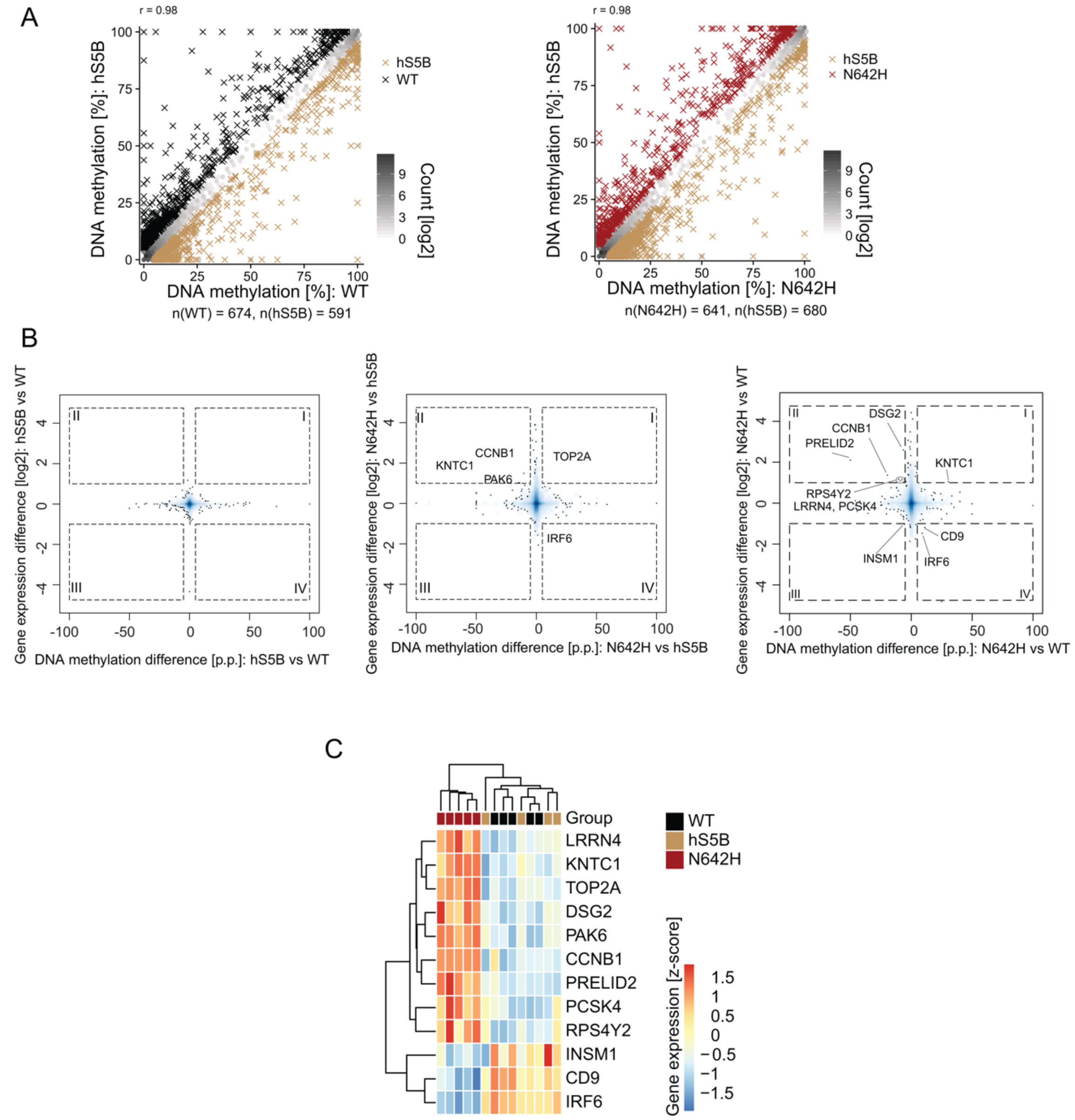

Supplemental Figure 8: DNA methylation pattern of CD8^{+} T-cells. (A) Scatterplot contrasting mean DNA methylation levels in WT vs hSTAT5B (hS5B) (left) and hSTAT5B vs hSTAT5B^{N642H} (N642H) (right) CD8^{+} T-cells in all CGIs covered in at least one sample per genotype (*n*=2/genotype). The density of data points in each plot region is indicated by color intensity (absolute difference ≥ 5 percentage points). **(B)** Density scatterplot contrasting hSTAT5B/WT or

hSTAT5B^{N642H}/hSTAT5B or hSTAT5B^{N642H}/WT differences in DNA methylation at CGIs proximal to transcription start sites (TSS, maximum distance = 10 kb) with expression differences of the corresponding genes. Genes with strong differences in DNA methylation (absolute difference ≥ 5 percentage points) and gene expression (|log2FC| ≥ 1) are highlighted. **(C)** Heat map visualizing the relative expression levels of twelve genes with strong differences in both DNA methylation and gene expression in all comparisons between WT, hSTAT5B, and hSTAT5B^{N642H} cells. Colors indicate expression levels and have been scaled such that red colors indicate the strongest and blue color the lowest expression per gene. Each column represents one dataset. Columns and rows have been rearranged by hierarchical clustering with complete linkage.

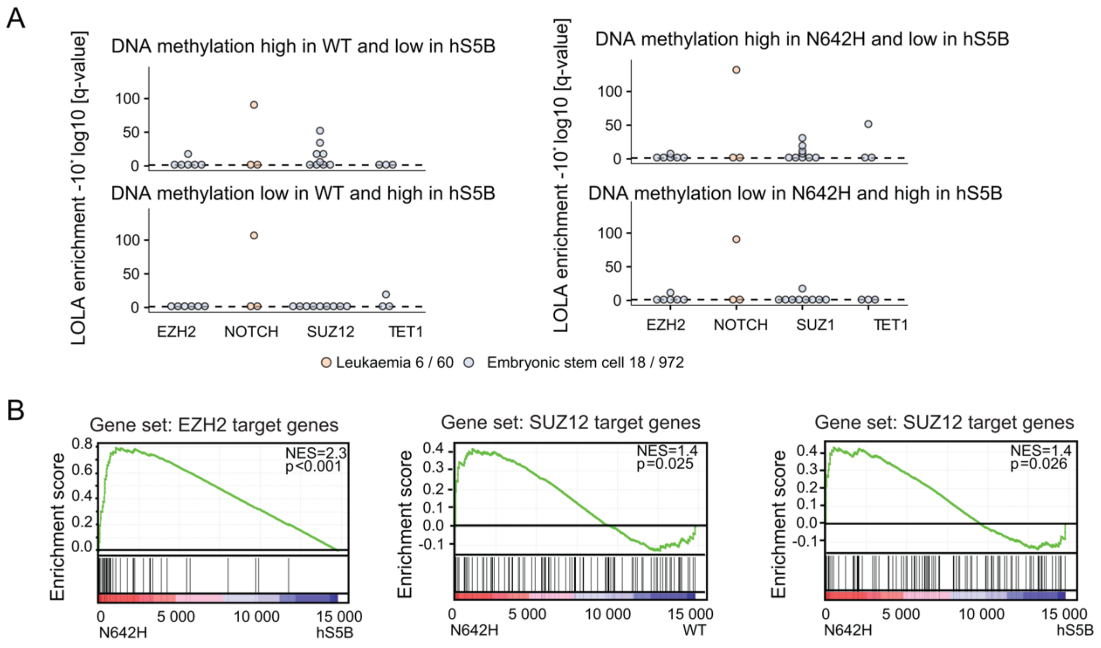

Supplemental Figure 9: DNA methylation status of specific binding sites. (A) Region set enrichment analysis for CGIs with lower DNA methylation in hSTAT5B (hS5B) than in WT (top left) or lower DNA methylation in WT than in hSTAT5B (bottom left) and for CGIs with lower DNA methylation in hSTAT5B than in hSTAT5B^{N642H} (top right) or lower DNA methylation in hSTAT5B^{N642H} than in hSTAT5B (bottom right). Enrichment was determined using Locus Overlap Analysis (LOLA). Each dot represents one ChIP-seq experiment for a given transcription factor from the CODEX database. The vertical dashed line represents the significance threshold (FDR-adjusted p-value ≤ 0.05). **(B)** Enrichment blot of EZH2 target genes in HSCs and SUZ12 target genes in embryonic stem cells. The barcode blot indicates the position of gene in the gene set. Red represents positive Pearson's correlation with hSTAT5B^{N642H} CD8^{+} T-cells and blue color represents positive Pearson's correlation with WT CD8^{+} T-cells. The gene set was obtained from Molecular signature database (1).

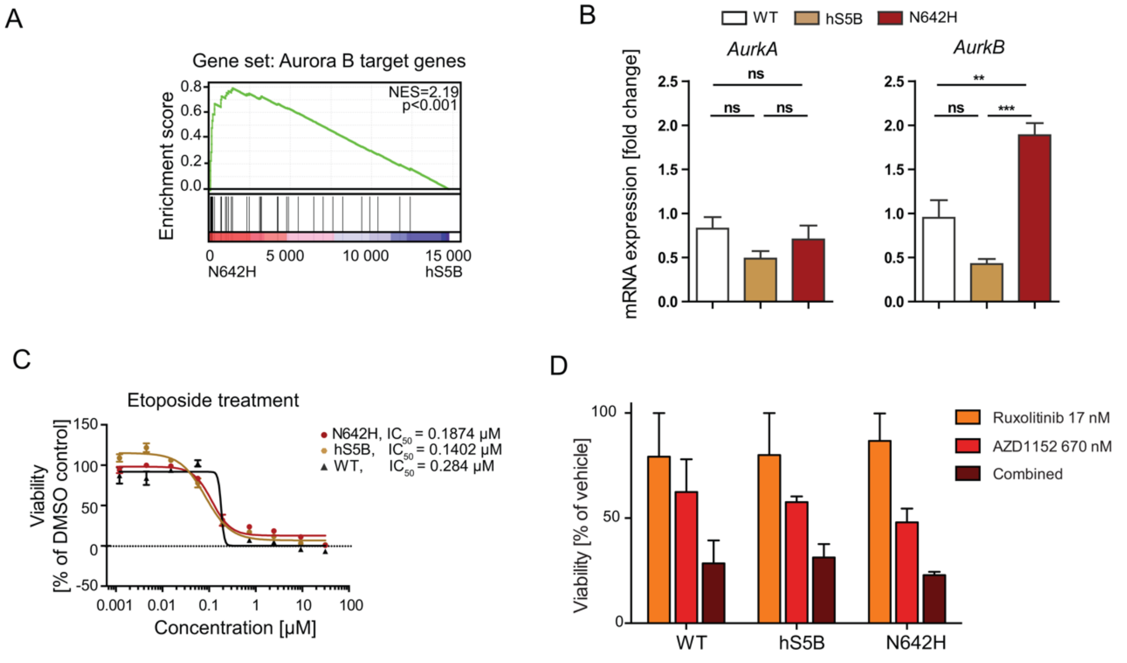

Supplemental Figure 10. **The enrichment of EZH2 target genes reveals novel targets for the treatment of hSTAT5B^{N642H}-driven disease. (A)** Enrichment blot of AURKB target genes. The barcode blot indicates the position of gene in the gene set. Red represents positive Pearson's correlation with hSTAT5B^{N642H} (N642H) CD8^{+} T-cells and blue color represents positive Pearson's correlation with WT CD8^{+} T-cells. The gene set was obtained from Molecular signature database (1). **(B)** mRNA expression of *AurkA* and *AurkB* from CD8^{+} T-cells of 13 week-old mice. $n \geq 3$. **(C)** Dose-response curve of WT T-cells and T-cells expressing hSTAT5B (hS5B) or hSTAT5B^{N642H} in response to 72h treatment with etoposide, TOP2A inhibitor analyzed using CTG assay (n=4 per genotype). IC_{50} values were determined using Graphpad Prism software. **(D)** Effect of low concentration of ruxolitinib in combination with AZD1152 on cell viability of WT T-cells and T-cells expressing hSTAT5B or hSTAT5B^{N642H} after 72h treatment,

analyzed using CTG assay. Error bars indicate ± SD. All data are presented as mean ± SD. *$P < 0.05$; **$P < 0.01$; ***$P < 0.001$; (1way ANOVA and Bonferroni corrected *P* value). Data presented in D is a representation of 2 independent experiments.

Supplemental Table 1: IC_{50} values of drugs tested on WT, hSTAT5B and hSTAT5B^{N642H} T-cells

Compound	IC_{50} [µM]		
	WT	STAT5B	STAT5B^{N642H}
Tofacitinib	0.24	0.1	0.12
Entinostat	0.24	0.59	0.49
JQ1	0.16	0.15	0.09
Etoposide	0.28	0.14	0.18
Ruxolitinib	0.21	0.16	0.11
5-Azacitidine	0.32	0.52	0.36

Supplemental Table 2: Top 50 up-regulated genes in hSTAT5B^{N642H}

	Average normalized fold of expression		
Gene name	WT	hSTAT5B	hSTAT5B^{N642H}
MYO6	-0.870653602	-0.498452588	1.269415672
FAT1	-0.761441206	-0.649424566	1.280980859
IGJ	-0.69783193	-0.724025384	1.277052237
GM1060	-0.992803179	-0.298555646	1.231647695
GZMB	-0.799522949	-0.596843026	1.27699737
IL12RB2	-0.764926485	-0.645588998	1.281397684
OSBPL3	-0.86526464	-0.504880599	1.269169119
FASL	-0.963219414	-0.349297197	1.242657172
AA467197	-0.693033977	-0.725862473	1.273723955
KIRREL	-0.820819737	-0.564862115	1.27270943
KLRC1	-0.909685102	-0.427346524	1.251562321

S100A6	-0.95569918	-0.351553249	1.236941779
GLDC	-0.688135612	-0.712748907	1.258334737
PIM1	-0.913018037	-0.426528614	1.254240928
SEPT8	-0.874618733	-0.481759321	1.26002619
LPIN1	-0.913210576	-0.424227733	1.252592763
GZMM	-0.984326233	-0.295293396	1.22056095
ENTPD1	-0.908631227	-0.427253114	1.250433718
LY6C1	-0.938540434	-0.138063695	1.04899139
CCR5	-0.974576774	-0.316160173	1.227504913
AQP9	-1.0079714	-0.259639493	1.215682994
LGALS3	-0.688118674	-0.713828978	1.259181857
STC2	-0.93104298	-0.39338042	1.245747316
PTGER3	-0.709555663	-0.698671591	1.268492936
IFNG	-0.78255179	-0.612404947	1.272475748
IFITM1	-0.675732458	-0.693844389	1.230807968
CCR2	-0.952146931	-0.344587153	1.227816654
RP23-320E6.1	-0.901587823	-0.432360979	1.247476607
CCNB2	-0.576418121	-0.839645961	1.248134889
D630039A03RIK	-0.669353607	-0.737911651	1.259682928
NM_010889	-0.895026601	-0.408847023	1.222104219
PSRC1	-0.771462836	-0.624335659	1.270931363
RCN1	-0.883898301	-0.46326955	1.254513941
CCL5	-0.919678005	-0.377836216	1.221946977
FAM19A3	-0.866826417	-0.484716546	1.254599655
F2R	-0.959269028	-0.338342192	1.229942782
MTAP6	-0.690826898	-0.726484031	1.272014123

KLRB1C	-0.703594545	-0.689476272	1.255175563
DYRK4	-0.940041418	-0.357057716	1.225687591
AC101743.4	-0.457475743	-0.930351737	1.201757133
5830411N06RIK	-0.910031966	-0.415371126	1.242328867
IL2RB	-0.96424611	-0.328061141	1.226695023
PRELID2	-0.674940544	-0.699792961	1.234774913
ZCCHC18	-0.903380784	-0.434500373	1.250981083
SKA1	-0.539026647	-0.85720637	1.224791743
CDC2A	-0.659246971	-0.733384018	1.245954185
SH3BGRL2	-0.564636582	-0.816520235	1.217852769
KLRE1	-0.907338358	-0.388287079	1.217968021
S1PR5	-0.812132194	-0.528598217	1.235010768
NPAS2	-0.756083451	-0.642495202	1.270079612

Supplemental Table 3: Top 50 down-regulated genes in hSTAT5B^{N642H}

	Average normalized fold of expression		
Gene name	WT	hSTAT5B	hSTAT5B^{N642H}
TMIE	0.801148288	0.584630342	-1.268852562
KCNA2	0.641371626	0.778150102	-1.263891708
IL6ST	0.777078692	0.6172273	-1.270860532
CNN3	0.861893931	0.502210608	-1.263662417
GPRC5B	0.771267267	0.618468046	-1.266041703
XKRX	0.853850414	0.505963655	-1.258621339
FMNL3	0.876210915	0.475863282	-1.256901541
CPM	0.798484096	0.581889559	-1.263995743

ENG	0.827986762	0.530785402	-1.252615083
CCR9	0.818778336	0.528045519	-1.241214752
TIMP2	0.742039526	0.651997767	-1.263637739
FAM101B	0.81248802	0.548576231	-1.251349005
KCNC1	0.790985068	0.577510822	-1.252993726
NTRK3	0.677983617	0.729401315	-1.261504669
CHST15	0.770815638	0.607903299	-1.257138278
ACTN1	0.77380593	0.603150473	-1.256326309
ST8SIA1	0.758460612	0.606813927	-1.243911754
IRF6	0.776274883	0.593290298	-1.250907121
MTSS1	0.827493473	0.524239538	-1.246885104
EPHX1	0.827379832	0.507024044	-1.232999068
BCL6	0.969071588	0.306283485	-1.214098376
ART2A	0.611444324	0.729630867	-1.195149018
TBC1D30	0.463861125	0.937481055	-1.213845969
LDLRAP1	0.775842197	0.594774477	-1.251661779
2610019F03RIK	0.689245969	0.690694299	-1.241801408
NAB2	0.815649746	0.517162277	-1.229379568
SEPP1	0.866029178	0.465859756	-1.238716983
MFHAS1	0.812477393	0.525113032	-1.232567819
MAML3	0.853499966	0.470462426	-1.229869907
E430029J22RIK	0.770945943	0.582827431	-1.237207888
AC159809.2	0.608972694	0.783133743	-1.235479689
CD209E	0.819305262	0.451555297	-1.1805495
CXXC5	0.919120177	0.372813547	-1.217371014
AFAP1	0.696071529	0.656611795	-1.221360965

ITGAE	0.936758427	0.338155214	-1.207282597
TLR12	0.811762638	0.524233903	-1.231149761
CYP2S1	0.503115697	0.874163952	-1.202446859
APP	0.728626442	0.620110438	-1.224714793
ART4	0.699393059	0.644005493	-1.214597453
GRIA3	0.829587886	0.485624957	-1.218087851
RNF19A	0.839272792	0.509928439	-1.247215543
CNGA1	0.815415353	0.511841031	-1.224888178
AMPD1	0.719700342	0.64504694	-1.235737894
PLEKHG2	0.705903026	0.64038242	-1.218208962
TSPAN9	0.99054511	0.244061536	-1.185794339
RGS10	0.733607925	0.60868815	-1.220558445
ABCA3	0.688468274	0.650657463	-1.208994244
MAP3K9	0.479833169	0.862278296	-1.169655806
NHSL1	0.924426576	0.358349035	-1.211105804
INADL	0.789685904	0.54395365	-1.224848824

Supplemental Table 4: LOLA analysis of DNA methylation

Differentially methylated regions	ChIP-seq reference data			Enrichment statistics				
User set	File name	Cell type	Antibody	Description	Log-odds	P-value	Q-value	-10*log10 (q-value)
DNA methylation high in hSTAT5B^{N642H} and low in hSTAT5B	GSM1014542_Ezh2.bed	Embryonic stem cell	Ezh2	N/A	1.30	0.07401	0.28575	5.4
	GSM327668_Ezh2.bed	Embryonic stem cell	Ezh2	N/A	1.28	0.22298	1.00000	0.0
	GSM905445_Ezh2.bed	Embryonic stem cell	Ezh2	129-C57Bl/6 ES cell differentiated *in vitro* by removal of LIF for 7 days	1.20	0.41673	1.00000	0.0
	GSM1215218_Ezh2.bed	Embryonic stem cell	Ezh2	C57BL/6-CBA ES cells	0.92	0.88092	1.00000	0.0
	GSM1059004_Ezh2.bed	Embryonic stem cell	Ezh2	ES cells expressing control shRNA targeting GFP	0.92	0.90285	1.00000	0.0
	GSM1199182_Ezh2.bed	Embryonic stem cell	Ezh2	Cells were lysed in ChIP buffer (50 mM Tris-HCl pH 7.9; 150 mM NaCl; 1% Triton X	0.69	1.00000	1.00000	0.0
	GSM859512_Notch1.bed	Leukemia	Notch1	intracellular Notch1-IC over-expressing tumors	2.68	0.00000	0.00000	132.1
	GSM732916_Notch1.bed	Leukemia	Notch1	NULL	0.42	0.99993	1.00000	0.0
	GSM732913_Notch1.bed	Leukemia	Notch1	NULL	0.29	1.00000	1.00000	0.0
	GSM1041374_Suz12.bed	Embryonic stem cell	Suz12	N/A	1.46	0.00365	0.00077	31.1
	GSM1019771_Suz12.bed	Embryonic stem cell	Suz12	N/A	1.38	0.01229	0.01118	19.5
	GSM1199188_Suz12.bed	Embryonic stem cell	Suz12	Cells were lysed in ChIP buffer (50 mM Tris-	1.34	0.03500	0.06856	11.6

				HCl pH 7.9; 150 mM NaCl; 1% Triton X				
	GSM1169018_Suz12.bed	Embryonic stem cell	Suz12	N/A	1.33	0.04232	0.09916	10.0
	GSM1169019_Suz12.bed	Embryonic stem cell	Suz12	N/A	1.27	0.08641	0.38215	4.2
	GSM700554_Suz12.bed	Embryonic stem cell	Suz12	ES cells expressing control shRNA	1.18	0.19905	1.00000	0.0
	GSM288360_Suz12.bed	Embryonic stem cell	Suz12	N/A	1.19	0.38450	1.00000	0.0
	GSM700553_Suz12_Pcl3_shRNA.bed	Embryonic stem cell	Suz12	ES cells expressing shRNA targeting Pcl3	1.08	0.49381	1.00000	0.0
	GSM307144_Suz12.bed	Embryonic stem cell	Suz12	N/A	0.90	0.87446	1.00000	0.0
	GSM611192_Tet1C.bed	Embryonic stem cell	Tet1	ES cell expressing scrabbled control shRNA. Antibody to Tet1 C-terminus	2.37	0.00039	0.00001	51.8
	GSM659799_Tet1.bed	Embryonic stem cell	Tet1	N/A	0.66	0.99483	1.00000	0.0
	GSM611194_Tet1N.bed	Embryonic stem cell	Tet1	ES cell expressing scrabbled control shRNA. Antibody to Tet1 N-terminus	0.41	1.00000	1.00000	0.0
DNA methylation low in STAT5B^{N642H} and high in hSTAT5B	GSM1014542_Ezh2.bed	Embryonic stem cell	Ezh2	N/A	1.35	0.03406	0.06856	11.6
	GSM327668_Ezh2.bed	Embryonic stem cell	Ezh2	N/A	1.35	0.13375	0.77539	1.1
	GSM905445_Ezh2.bed	Embryonic stem cell	Ezh2	129-C57Bl/6 ES cell differentiated *in vitro* by removal of LIF for 7 days	1.33	0.21238	1.00000	0.0
	GSM1215218_Ezh2.bed	Embryonic stem cell	Ezh2	C57BL/6-CBA ES cells	1.07	0.59011	1.00000	0.0
	GSM1059004_Ezh2.bed	Embryonic stem cell	Ezh2	ES cells expressing control shRNA targeting GFP	1.02	0.71316	1.00000	0.0
	GSM1199182_Ezh2.bed	Embryonic stem cell	Ezh2	Cells were lysed in ChIP buffer (50 mM Tris-HCl pH 7.9; 150 mM NaCl; 1% Triton X	0.67	1.00000	1.00000	0.0
	GSM859512_Notch1.bed	Leukemia	Notch1	intracellular Notch1-IC over-expressing tumors	2.28	0.00001	0.00000	90.9

	GSM732916_Notch1.bed	Leukemia	Notch1	NULL	0.30	1.00000	1.00000	0.0
	GSM732913_Notch1.bed	Leukemia	Notch1	NULL	0.21	1.00000	1.00000	0.0
	GSM1041374_Suz12.bed	Embryonic stem cell	Suz12	N/A	1.36	0.01595	0.01667	17.8
	GSM700554_Suz12.bed	Embryonic stem cell	Suz12	ES cells expressing control shRNA	1.22	0.10682	0.54192	2.7
	GSM1019771_Suz12.bed	Embryonic stem cell	Suz12	N/A	1.21	0.11564	0.58958	2.3
	GSM1169019_Suz12.bed	Embryonic stem cell	Suz12	N/A	1.22	0.14795	0.95314	0.2
	GSM307144_Suz12.bed	Embryonic stem cell	Suz12	N/A	1.43	0.16882	1.00000	0.0
	GSM700553_Suz12_Pcl3_shRNA.bed	Embryonic stem cell	Suz12	ES cells expressing shRNA targeting Pcl3	1.19	0.19374	1.00000	0.0
	GSM1169018_Suz12.bed	Embryonic stem cell	Suz12	N/A	1.17	0.23296	1.00000	0.0
	GSM288360_Suz12.bed	Embryonic stem cell	Suz12	N/A	1.27	0.23880	1.00000	0.0
	GSM1199188_Suz12.bed	Embryonic stem cell	Suz12	Cells were lysed in ChIP buffer (50 mM Tris-HCl pH 7.9; 150 mM NaCl; 1% Triton X	1.13	0.32472	1.00000	0.0
	GSM611192_Tet1C.bed	Embryonic stem cell	Tet1	ES cell expressing scrabbled control shRNA. Antibody to Tet1 C-terminus	1.42	0.19500	1.00000	0.0
	GSM659799_Tet1.bed	Embryonic stem cell	Tet1	N/A	0.53	0.99980	1.00000	0.0
	GSM611194_Tet1N.bed	Embryonic stem cell	Tet1	ES cell expressing scrabbled control shRNA. Antibody to Tet1 N-terminus	0.38	1.00000	1.00000	0.0
DNA methylation high in WT and low STAT5B^{N642H}	GSM1014542_Ezh2.bed	Embryonic stem cell	Ezh2	N/A	1.49	0.00257	0.00036	34.4
	GSM905445_Ezh2.bed	Embryonic stem cell	Ezh2	129-C57Bl/6 ES cell differentiated *in vitro* by removal of LIF for 7 days	1.58	0.03116	0.05913	12.3
	GSM327668_Ezh2.bed	Embryonic stem cell	Ezh2	N/A	1.43	0.05729	0.17878	7.5
	GSM1215218_Ezh2.bed	Embryonic stem cell	Ezh2	C57BL/6-CBA ES cells	1.15	0.38742	1.00000	0.0

	GSM1059004_Ezh2.bed	Embryonic stem cell	Ezh2	ES cells expressing control shRNA targeting GFP	1.04	0.64442	1.00000	0.0
	GSM1199182_Ezh2.bed	Embryonic stem cell	Ezh2	Cells were lysed in ChIP buffer (50 mM Tris-HCl pH 7.9; 150 mM NaCl; 1% Triton X	0.77	0.99993	1.00000	0.0
	GSM859512_Notch1.bed	Leukemia	Notch1	intracellular Notch1-IC over-expressing tumors	2.46	0.00000	0.00000	126.4
	GSM732916_Notch1.bed	Leukemia	Notch1	NULL	0.29	1.00000	1.00000	0.0
	GSM732913_Notch1.bed	Leukemia	Notch1	NULL	0.21	1.00000	1.00000	0.0
	GSM1041374_Suz12.bed	Embryonic stem cell	Suz12	N/A	1.61	0.00005	0.00000	71.1
	GSM1019771_Suz12.bed	Embryonic stem cell	Suz12	N/A	1.50	0.00041	0.00001	51.7
	GSM700554_Suz12.bed	Embryonic stem cell	Suz12	ES cells expressing control shRNA	1.47	0.00087	0.00003	44.6
	GSM1169019_Suz12.bed	Embryonic stem cell	Suz12	N/A	1.35	0.01934	0.02382	16.2
	GSM700553_Suz12_Pcl3_shRNA.bed	Embryonic stem cell	Suz12	ES cells expressing shRNA targeting Pcl3	1.33	0.02358	0.03473	14.6
	GSM1169018_Suz12.bed	Embryonic stem cell	Suz12	N/A	1.33	0.02696	0.04389	13.6
	GSM1199188_Suz12.bed	Embryonic stem cell	Suz12	Cells were lysed in ChIP buffer (50 mM Tris-HCl pH 7.9; 150 mM NaCl; 1% Triton X	1.29	0.04384	0.10459	9.8
	GSM288360_Suz12.bed	Embryonic stem cell	Suz12	N/A	1.28	0.20392	1.00000	0.0
	GSM307144_Suz12.bed	Embryonic stem cell	Suz12	N/A	1.18	0.47422	1.00000	0.0
	GSM611192_Tet1C.bed	Embryonic stem cell	Tet1	ES cell expressing scrabbled control shRNA. Antibody to Tet1 C-terminus	1.32	0.27642	1.00000	0.0
	GSM659799_Tet1.bed	Embryonic stem cell	Tet1	N/A	0.65	0.99753	1.00000	0.0
	GSM611194_Tet1N.bed	Embryonic stem cell	Tet1	ES cell expressing scrabbled control shRNA. Antibody to Tet1 N-terminus	0.38	1.00000	1.00000	0.0

DNA methylation high in $hSTAT5B^{N642H}$ and low in WT	GSM327668_Ezh2.bed	Embryonic stem cell	Ezh2	N/A	1.16	0.43134	1.00000	0.0
	GSM1014542_Ezh2.bed	Embryonic stem cell	Ezh2	N/A	0.99	0.76311	1.00000	0.0
	GSM905445_Ezh2.bed	Embryonic stem cell	Ezh2	129-C57Bl/6 ES cell differentiated *in vitro* by removal of LIF for 7 days	0.80	0.96168	1.00000	0.0
	GSM1215218_Ezh2.bed	Embryonic stem cell	Ezh2	C57BL/6-CBA ES cells	0.71	0.99682	1.00000	0.0
	GSM1059004_Ezh2.bed	Embryonic stem cell	Ezh2	ES cells expressing control shRNA targeting GFP	0.66	0.99985	1.00000	0.0
	GSM1199182_Ezh2.bed	Embryonic stem cell	Ezh2	Cells were lysed in ChIP buffer (50 mM Tris-HCl pH 7.9; 150 mM NaCl; 1% Triton X	0.58	1.00000	1.00000	0.0
	GSM859512_Notch1.bed	Leukemia	Notch1	intracellular Notch1-IC over-expressing tumors	2.63	0.00000	0.00000	123.1
	GSM732916_Notch1.bed	Leukemia	Notch1	NULL	0.44	0.99983	1.00000	0.0
	GSM732913_Notch1.bed	Leukemia	Notch1	NULL	0.27	1.00000	1.00000	0.0
	GSM1041374_Suz12.bed	Embryonic stem cell	Suz12	N/A	1.18	0.22017	1.00000	0.0
	GSM1169019_Suz12.bed	Embryonic stem cell	Suz12	N/A	1.17	0.27316	1.00000	0.0
	GSM1169018_Suz12.bed	Embryonic stem cell	Suz12	N/A	1.14	0.33296	1.00000	0.0
	GSM1019771_Suz12.bed	Embryonic stem cell	Suz12	N/A	1.12	0.35377	1.00000	0.0
	GSM288360_Suz12.bed	Embryonic stem cell	Suz12	N/A	1.06	0.64628	1.00000	0.0
	GSM1199188_Suz12.bed	Embryonic stem cell	Suz12	Cells were lysed in ChIP buffer (50 mM Tris-HCl pH 7.9; 150 mM NaCl; 1% Triton X	1.03	0.66013	1.00000	0.0
	GSM307144_Suz12.bed	Embryonic stem cell	Suz12	N/A	1.02	0.73098	1.00000	0.0
	GSM700553_Suz12_Pcl3_shRNA.bed	Embryonic stem cell	Suz12	ES cells expressing shRNA targeting Pcl3	0.97	0.81911	1.00000	0.0
	GSM700554_Suz12.bed	Embryonic stem cell	Suz12	ES cells expressing control shRNA	0.97	0.83610	1.00000	0.0

	GSM611192_Tet1C.bed	Embryonic stem cell	Tet1	ES cell expressing scrabbled control shRNA. Antibody to Tet1 C-terminus	2.60	0.00008	0.00000	66.7
	GSM659799_Tet1.bed	Embryonic stem cell	Tet1	N/A	0.72	0.98348	1.00000	0.0
	GSM611194_Tet1N.bed	Embryonic stem cell	Tet1	ES cell expressing scrabbled control shRNA. Antibody to Tet1 N-terminus	0.40	1.00000	1.00000	0.0
DNA methylation high in WT and low in hSTAT5B	GSM1014542_Ezh2.bed	Embryonic stem cell	Ezh2	N/A	1.43	0.01267	0.01136	19.4
	GSM905445_Ezh2.bed	Embryonic stem cell	Ezh2	129-C57Bl/6 ES cell differentiated *in vitro* by removal of LIF for 7 days	1.47	0.09176	0.42086	3.8
	GSM327668_Ezh2.bed	Embryonic stem cell	Ezh2	N/A	1.26	0.24275	1.00000	0.0
	GSM1059004_Ezh2.bed	Embryonic stem cell	Ezh2	ES cells expressing control shRNA targeting GFP	1.05	0.61359	1.00000	0.0
	GSM1215218_Ezh2.bed	Embryonic stem cell	Ezh2	C57BL/6-CBA ES cells	0.99	0.77329	1.00000	0.0
DNA methylation high in WT and low in hSTAT5B	GSM1199182_Ezh2.bed	Embryonic stem cell	Ezh2	Cells were lysed in ChIP buffer (50 mM Tris-HCl pH 7.9; 150 mM NaCl; 1% Triton X	0.72	1.00000	1.00000	0.0
	GSM859512_Notch1.bed	Leukemia	Notch1	intracellular Notch1-IC over-expressing tumors	2.29	0.00001	0.00000	90.6
	GSM732916_Notch1.bed	Leukemia	Notch1	NULL	0.40	0.99998	1.00000	0.0
	GSM732913_Notch1.bed	Leukemia	Notch1	NULL	0.27	1.00000	1.00000	0.0
	GSM1041374_Suz12.bed	Embryonic stem cell	Suz12	N/A	1.57	0.00036	0.00001	52.3
	GSM1019771_Suz12.bed	Embryonic stem cell	Suz12	N/A	1.45	0.00254	0.00036	34.4
	GSM1169018_Suz12.bed	Embryonic stem cell	Suz12	N/A	1.38	0.01846	0.02235	16.5
	GSM1199188_Suz12.bed	Embryonic stem cell	Suz12	Cells were lysed in ChIP buffer (50 mM Tris-HCl pH 7.9; 150 mM NaCl; 1% Triton X	1.36	0.02015	0.02516	16.0

	GSM700554_Suz12.bed	Embryonic stem cell	Suz12	ES cells expressing control shRNA	1.25	0.06840	0.25574	5.9
	GSM288360_Suz12.bed	Embryonic stem cell	Suz12	N/A	1.32	0.17404	1.00000	0.0
	GSM700553_Suz12_Pcl3_shRNA.bed	Embryonic stem cell	Suz12	ES cells expressing shRNA targeting Pcl3	1.20	0.17562	1.00000	0.0
	GSM1169019_Suz12.bed	Embryonic stem cell	Suz12	N/A	1.15	0.29485	1.00000	0.0
	GSM307144_Suz12.bed	Embryonic stem cell	Suz12	N/A	1.08	0.63973	1.00000	0.0
	GSM611192_Tet1C.bed	Embryonic stem cell	Tet1	ES cell expressing scrabbled control shRNA. Antibody to Tet1 C-terminus	1.53	0.11509	0.58958	2.3
	GSM659799_Tet1.bed	Embryonic stem cell	Tet1	N/A	0.70	0.99088	1.00000	0.0
	GSM611194_Tet1N.bed	Embryonic stem cell	Tet1	ES cell expressing scrabbled control shRNA. Antibody to Tet1 N-terminus	0.40	1.00000	1.00000	0.0
DNA methylation high in STAT5B and low in WT	GSM1014542_Ezh2.bed	Embryonic stem cell	Ezh2	N/A	1.23	0.17368	1.00000	0.0
	GSM327668_Ezh2.bed	Embryonic stem cell	Ezh2	N/A	1.24	0.30004	1.00000	0.0
	GSM905445_Ezh2.bed	Embryonic stem cell	Ezh2	129-C57Bl/6 ES cell differentiated *in vitro* by removal of LIF for 7 days	0.90	0.88413	1.00000	0.0
	GSM1059004_Ezh2.bed	Embryonic stem cell	Ezh2	ES cells expressing control shRNA targeting GFP	0.86	0.95805	1.00000	0.0
	GSM1215218_Ezh2.bed	Embryonic stem cell	Ezh2	C57BL/6-CBA ES cells	0.83	0.96337	1.00000	0.0
	GSM1199182_Ezh2.bed	Embryonic stem cell	Ezh2	Cells were lysed in ChIP buffer (50 mM Tris-HCl pH 7.9; 150 mM NaCl; 1% Triton X	0.62	1.00000	1.00000	0.0
	GSM859512_Notch1.bed	Leukemia	Notch1	intracellular Notch1-IC over-expressing tumors	2.52	0.00000	0.00000	106.9
	GSM732916_Notch1.bed	Leukemia	Notch1	NULL	0.38	0.99996	1.00000	0.0
	GSM732913_Notch1.bed	Leukemia	Notch1	NULL	0.28	1.00000	1.00000	0.0

	GSM1041374_Suz12.bed	Embryonic stem cell	Suz12	N/A	1.26	0.09065	0.41779	3.8
	GSM1169019_Suz12.bed	Embryonic stem cell	Suz12	N/A	1.22	0.17392	1.00000	0.0
	GSM1169018_Suz12.bed	Embryonic stem cell	Suz12	N/A	1.19	0.22200	1.00000	0.0
	GSM1019771_Suz12.bed	Embryonic stem cell	Suz12	N/A	1.14	0.30745	1.00000	0.0
	GSM1199188_Suz12.bed	Embryonic stem cell	Suz12	Cells were lysed in ChIP buffer (50 mM Tris-HCl pH 7.9; 150 mM NaCl; 1% Triton X	1.13	0.35967	1.00000	0.0
	GSM307144_Suz12.bed	Embryonic stem cell	Suz12	N/A	1.20	0.47061	1.00000	0.0
	GSM700553_Suz12_Pcl3_shRNA.bed	Embryonic stem cell	Suz12	ES cells expressing shRNA targeting Pcl3	1.07	0.54624	1.00000	0.0
	GSM700554_Suz12.bed	Embryonic stem cell	Suz12	ES cells expressing control shRNA	1.06	0.54898	1.00000	0.0
	GSM288360_Suz12.bed	Embryonic stem cell	Suz12	N/A	1.09	0.59090	1.00000	0.0
	GSM611192_Tet1C.bed	Embryonic stem cell	Tet1	ES cell expressing scrabbled control shRNA. Antibody to Tet1 C-terminus	1.94	0.01297	0.01140	19.4
	GSM659799_Tet1.bed	Embryonic stem cell	Tet1	N/A	0.51	0.99975	1.00000	0.0
	GSM611194_Tet1N.bed	Embryonic stem cell	Tet1	ES cell expressing scrabbled control shRNA. Antibody to Tet1 N-terminus	0.36	1.00000	1.00000	0.0

Supplemental Table 5: DNA methylation status at EZH2 binding site

Chromosom	Start	End	Target gene	Differentially methylated	EZH2 target
chr1	20819889	20820297	MCM3	0.081994315	Not bound
chr1	20890435	20890811	MCM3	1.911259612	Not bound
chr1	191821250	191821971	NEK2	-0.289590773	Bound
chr1	191906597	191907697	NEK2	-0.079683446	Not bound
chr2	71367519	71367773	HAT1	-0.328074202	Not bound
chr2	71389037	71389586	HAT1	-0.712346064	Not bound
chr2	71453142	71453517	HAT1	-0.555555556	Not bound
chr2	72476060	72477161	CDCA7	-1.921243901	Not bound
chr2	119546917	119547155	NUSAP1	4.857024525	Not bound
chr2	119547472	119547988	NUSAP1	0.175233943	Bound
chr2	119594232	119594717	NUSAP1	-1.532116121	Not bound
chr2	119618173	119618652	NUSAP1	0	Not bound
chr2	119662622	119662879	NUSAP1	0.146942264	Bound
chr2	119674934	119675628	NUSAP1	1.667495566	Not bound
chr2	132247528	132247857	PCNA	1.146266353	Not bound
chr2	132262954	132263752	PCNA	0.04653081	Not bound
chr2	155074085	155074476	AHCY	1.823727139	Bound
chr2	155133431	155133879	AHCY	0.297424093	Bound
chr2	172345330	172345900	AURKA	-1.966563736	Not bound
chr2	172370597	172371333	AURKA	-0.615172595	Not bound
chr2	172440317	172440790	AURKA	0.631820639	Not bound
chr3	27153414	27154068	ECT2	0.583915529	Not bound
chr3	27182878	27183286	ECT2	-1.498472116	Not bound
chr3	137864038	137865781	H2AFZ	-1.840631785	Not bound
chr3	137867582	137868040	H2AFZ	0.286924613	Not bound
chr3	137918409	137918684	H2AFZ	0.313627234	Not bound
chr5	33629016	33629950	TACC3	-0.332267772	Not bound
chr5	33651872	33652808	TACC3	0.297619048	Not bound
chr5	33657578	33658835	TACC3	-0.330915718	Bound

chr5	33692519	33692773	TACC3	7.400055752	Not bound
chr5	33695268	33695842	TACC3	0.807956618	Bound
chr5	33721593	33723909	TACC3	-0.029648768	Bound
chr6	8209078	8209710	RPA3	0.447874045	Not bound
chr6	8258939	8259647	RPA3	-0.452192735	Not bound
chr6	66535130	66535719	MAD2L1	-2.26007642	Not bound
chr6	88841539	88843023	MCM2	0.434944866	Not bound
chr6	88874242	88875232	MCM2	-10.25249892	Bound
chr6	88898587	88898835	MCM2	1.445297667	Not bound
chr6	88901959	88902760	MCM2	0.623889341	Not bound
chr7	80198190	80198815	PRC1	-0.101163361	Bound
chr7	80232412	80232922	PRC1	-0.614239503	Not bound
chr7	80246218	80246618	PRC1	-0.248772244	Not bound
chr7	80261133	80261518	PRC1	-1.53710692	Not bound
chr7	80269579	80269844	PRC1	1.318083344	Not bound
chr7	80294257	80294862	PRC1	0	Not bound
chr7	80324099	80324365	PRC1	2.083333333	Not bound
chr7	80339932	80340448	PRC1	-0.83105039	Not bound
chr7	80363080	80363296	PRC1	0	Not bound
chr7	80370617	80371559	PRC1	0.620695746	Bound
chr7	102441613	102442102	RRM1	1.413637614	Bound
chr7	102512908	102513374	RRM1	0.4135878	Bound
chr8	13287140	13288476	TFDP1	0.736707017	Bound
chr8	13338535	13339396	TFDP1	0.594743839	Not bound
chr9	70421361	70421597	CCNB2	0.266193638	Not bound
chr9	70503067	70503877	CCNB2	-0.128997261	Not bound
chr10	111472969	111473826	NAP1L1	0.104891576	Not bound
chr10	111506138	111506897	NAP1L1	-0.435992834	Not bound
chr11	68967718	68968283	AURKB	-0.82577107	Not bound
chr11	69039957	69040362	AURKB	0.617360508	Not bound
chr11	69045641	69046110	AURKB	-0.46107311	Not bound
chr11	69059294	69060850	AURKB	-1.197594644	Not bound
chr11	69070707	69071062	AURKB	-0.820660934	Not bound

chr11	69088414	69089149	AURKB	-0.631137376	Not bound
chr11	69095157	69095360	AURKB	1.291666667	Not bound
chr11	69117978	69118214	AURKB	2.490329453	Not bound
chr11	69122149	69123102	AURKB	0.52396829	Bound
chr11	98936172	98936439	TOP2A	6.193568336	Bound
chr11	98937355	98938138	TOP2A	1.267700759	Bound
chr11	98959605	98960112	TOP2A	-0.884518404	Bound
chr11	98960435	98961001	TOP2A	0.334593987	Not bound
chr11	98982169	98983029	TOP2A	-1.141416983	Bound
chr11	99023930	99024182	TOP2A	4.232500768	Not bound
chr11	99041012	99041815	TOP2A	2.740718795	Bound
chr11	106920094	106920955	KPNA2	-0.066803139	Not bound
chr11	106999065	106999677	KPNA2	-2.528131486	Not bound
chr11	107012601	107012831	KPNA2	-1.160322682	Not bound
chr11	107027746	107028594	KPNA2	-0.524302036	Not bound
chr13	24761560	24762170	GMNN	-0.714749523	Not bound
chr13	24801094	24801950	GMNN	-0.298877611	Not bound
chr13	24802080	24802349	GMNN	-0.130779796	Not bound
chr13	24831403	24832195	GMNN	0.154445991	Not bound
chr13	24845055	24845340	GMNN	-1.067821068	Not bound
chr19	6015454	6015722	CDCA5	-0.561326523	Not bound
chr19	6046737	6047482	CDCA5	0.58846546	Not bound
chr19	6057666	6058189	CDCA5	-0.137362637	Not bound
chr19	6061019	6061919	CDCA5	-0.06902404	Not bound
chr19	6084755	6085356	CDCA5	1.516116932	Not bound
chr19	6117859	6118613	CDCA5	1.395758731	Bound
chr19	6140555	6141406	CDCA5	-2.048640416	Not bound
chr19	6184251	6184665	CDCA5	4.63911381	Not bound

Supplemental Table 6: Antibodies for flow cytometry

Antigen	Flourochrome	Clone	Catalog No
Fc-Block™	Unconjugated	93	14-0161-82 (ebioscience)
CD3	eFluor® 450	17A2	48-0032-82 (ebioscience)
CD3e	PerCP Cy5.5	145-2C11	45-0031-82 (ebioscience)
CD19	PE	eBio1D3 (1D3)	12-0193-82 (ebioscience)
CD19	eFluor® 450	eBio1D3 (1D3)	48-0193-82 (ebioscience)
CD4	FITC	GK1.5	11-0041-82 (ebioscience)
CD4	PE	GK1.5	12-0041-82 (ebioscience)
CD8a	FITC	53-6.7	11-0081-82 (ebioscience)
CD8a	PE	53-6.7	12-0081-82 (ebioscience)
CD11b	FITC	M1/70	11-0112-82 (ebioscience)
CD11b	eFluor® 450	M1/70	48-0112-82 (ebioscience)
CD25	APC	PC61.5	17-0251-81 (ebioscience)
CD44	PE	IM7	12-0441-82 (ebioscience)
CD117 (c-Kit)	PE-Cy5	2B8	15-1171-83 (ebioscience)
CD45.1 (Ly5.1)	PE	A20	12-0453-82 (ebioscience)
CD45.2 (Ly5.2)	APC	104	17-0454-82 (ebioscience)
CD45R (B220)	eFluor® 450	RA3-6B2	48-0452-82 (ebioscience)
CD45R (B220)	PerCP Cy5.5	RA3-6B2	45-0452-82 (ebioscience)

Ly6.G (Gr1)	APC	RB6-8C5	17-5931-82 (ebioscience)
Ly6.G (Gr1)	eFluor® 450	RB6-8C5	48-5931-82 (ebioscience)
Ly-6A/E (Sca-1)	PE-Cy7	D7	25-5981-82 (ebioscience)
Strepdavidin	APC-eFluor® 780		47-4317-82 (ebioscience)
TER-119	eFluor 450	TER-119	48-5921-82 (ebioscience)
CD62L	APC	MEL-14	17-0621-81 (ebioscience)
NK1.1	PE-Cyanine7	PK136	25-5941-82 (ebioscience)
CD135	Unconjugated	A2F10.1	13-1351-82 (ebioscience)
CD127	Unconjugated	A7R34	13-1271-82 (ebioscience)
CD16/CD32	PE	93	12-0161-82 (ebioscience)
CD93 (AA4.1)	APC	AA4.1	17-5892-82 (ebioscience)
Hamster anti-Mouse CD3e	Unconjugated	145-2C11	51-01082J (BD)
Rat anti-Mouse CD45R	Unconjugated	RA3-6B2	51-01122J (BD)
Rat anti-Mouse Ly-6G/C	Unconjugated	RB6-8C5	51-01212J (BD)
Rat anti-Mouse CD11b	Unconjugated	M1/70	51-01712J (BD)
Rat anti-Mouse TER-119	Unconjugated	TER-119	51-09082J (BD)
CD34	FITC	RAM34	553733 (BD)
CD150	APC	TC15-12F12.2	562373 (BD)
CD48	PE	HM48-1	557484 (BD)
CD34	APC	MEC14.7	119310 (Biolegend)

Supplemental Table 7: qPCR primers

	Forward primer	Reverse primer
Bcl2	GGTCTTCAGAGACAGCCAGG	GATCCAGGATAACGGAGGCT
Pim1	TGTTCTCGTCCTTGATGTCG	GGAGCCCTACAGGAGGACC
Aurk a	CAATGCTTGCTGACAGACACCCC	CACAGGCAGGAACCTGCTCC
Aurk b	ACGCGTAAATGCACACAAGCCCTG	AGATCTAGCTGGACAGAGAGG
Negative region	TACCCCTTCCAACTCTGACTGAGC	TTCCCTCCAGGATGTGACTGTG
P.R.*Ccnd2*	TCAACACGTGGTCAGAGTGG	GGGAAAGTTAAAGCGCGTCC
P.R.*Cis*	GTCCAAAGCACTAGACGCCTG	TTCCCGGAAGCCTCATCTT
P.R. *Cdkn2a*	TGTACAGAATCCTAGCACTGATACAGCAAC	GATCCCAACAACCCTAGCTCAAACAAC
CpGi *Aurb-01*	TAAGCATTCTGTACCTGTGTCC	GTGGAGAAGAGCAAGTTCTGA
CpGi *Aurb-02*	CTGCGTCAGTTTCCCACTTG	GAGAGGTGTACGAAACAGCAAC

Reference:

1. Kamminga, L.M., Bystrykh, L.V., de Boer, A., Houwer, S., Douma, J., Weersing, E., Dontje, B., and de Haan, G. 2006. The Polycomb group gene Ezh2 prevents hematopoietic stem cell exhaustion. *Blood* 107:2170-2179.

Full unedited blots for figure 1B

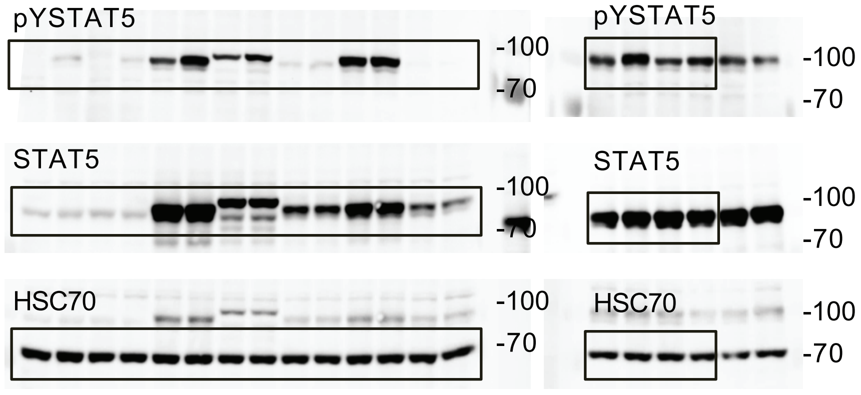

Full unedited blots for figure 1C

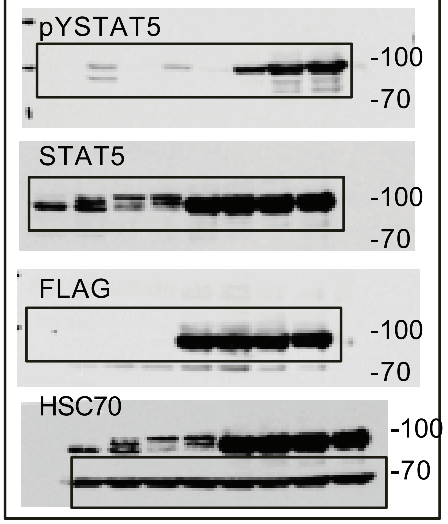

Full unedited blots for figure 2B

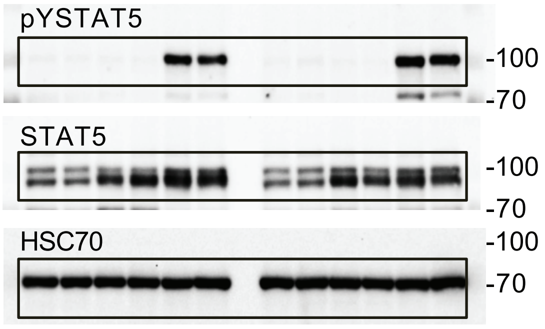

Full unedited blots for figure 6A

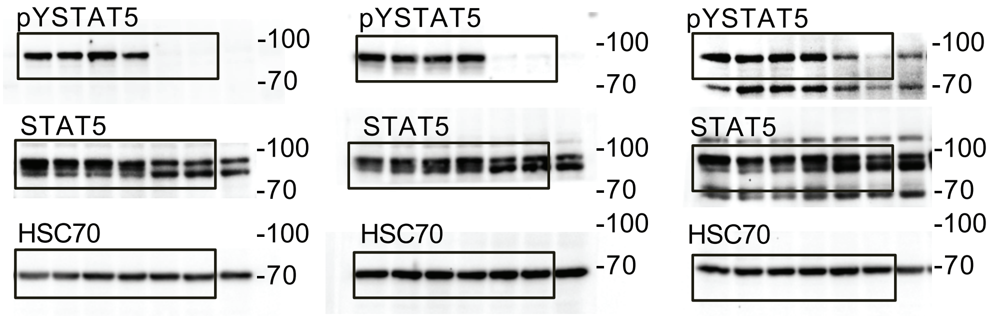

Full unedited blots for figure 6C

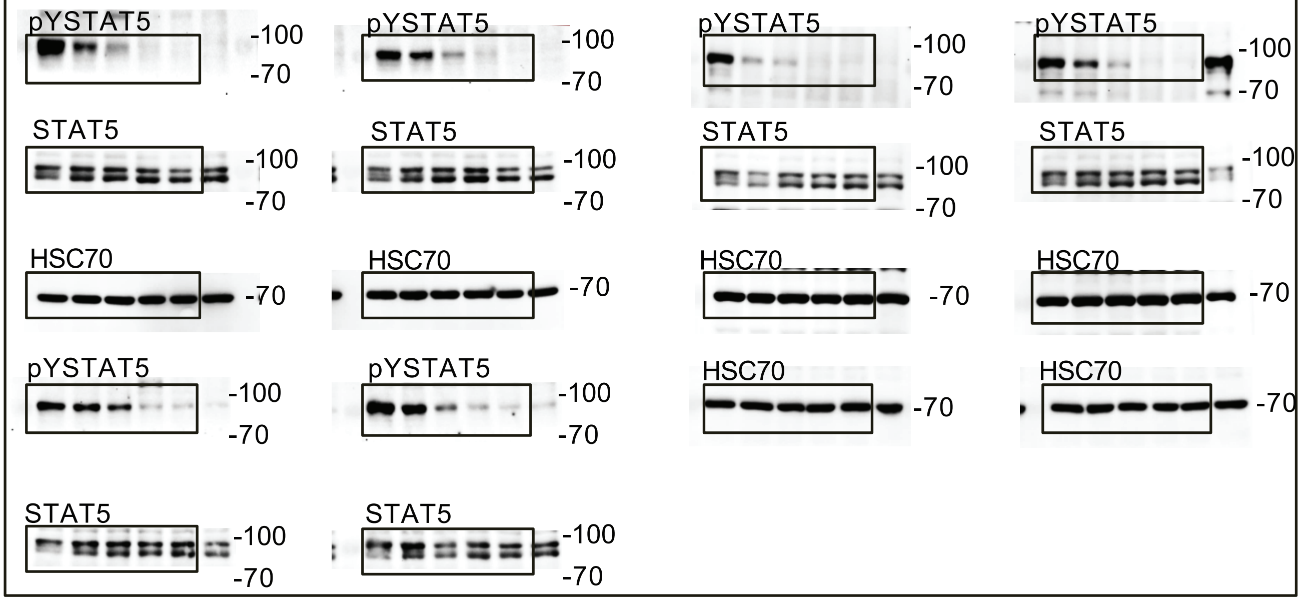

Supplement Figure 12

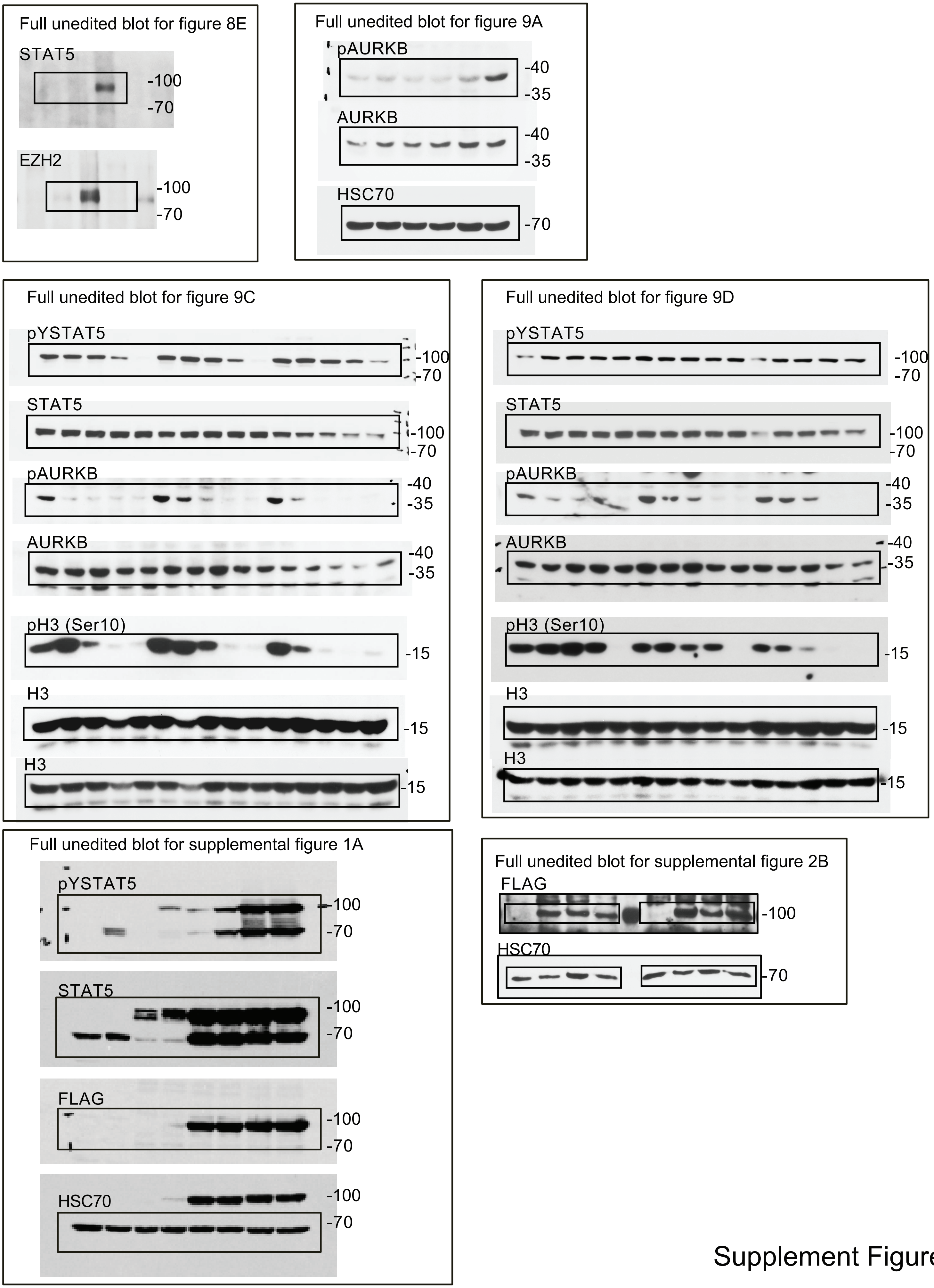

Supplement Figure 12
